# Dynamics and functional interplay of histone lysine butyrylation, crotonylation, and acetylation in rice under starvation and submergence

**DOI:** 10.1186/s13059-018-1533-y

**Published:** 2018-09-25

**Authors:** Yue Lu, Qiutao Xu, Yuan Liu, Yue Yu, Zhong-Yi Cheng, Yu Zhao, Dao-Xiu Zhou

**Affiliations:** 1 National Key Laboratory of Crop Genetic Improvement, Huazhong Agricultural University, Wuhan, 430070 China; 2 Institute of Plant Science of Paris-Saclay (IPS2), CNRS, INRA, University Paris-sud 11, University Paris-Saclay, 91405 Orsay, France; 3 Jingjie PTM BioLab Co. Ltd, Hangzhou, 310018 China

## Abstract

**Background:**

Histone lysine acylations by short-chain fatty acids are distinct from the widely studied histone lysine acetylation in chromatin, although both modifications are regulated by primary metabolism in mammalian cells. It remains unknown whether and how histone acylation and acetylation interact to regulate gene expression in plants that have distinct regulatory pathways of primary metabolism.

**Results:**

We identify 4 lysine butyrylation (Kbu) sites (H3K14, H4K12, H2BK42, and H2BK134) and 45 crotonylation (Kcr) sites on rice histones by mass spectrometry. Comparative analysis of genome-wide Kbu and Kcr and H3K9ac in combination with RNA sequencing reveals 25,306 genes marked by Kbu and Kcr in rice and more than 95% of H3K9ac-marked genes are marked by both. Kbu and Kcr are enriched at the 5′ region of expressed genes. In rice under starvation and submergence, Kbu and Kcr appear to be less dynamic and display changes in different sets of genes compared to H3K9ac. Furthermore, Kbu seems to preferentially poise gene activation by external stresses, rather than internal circadian rhythm which has been shown to be tightly associated with H3K9ac. In addition, we show that rice sirtuin histone deacetylase (SRT2) is involved in the removal of Kcr.

**Conclusion:**

Kbu, Kcr, and H3K9ac redundantly mark a large number of active genes but display different responses to external and internal signals. Thus, the proportion of rice histone lysine acetylation and acylation is dynamically regulated by environmental and metabolic cues, which may represent an epigenetic mechanism to fine-tune gene expression for plant adaptation.

**Electronic supplementary material:**

The online version of this article (10.1186/s13059-018-1533-y) contains supplementary material, which is available to authorized users.

## Background

Post-translational modifications of histones affect chromatin state and gene expression. For example, histone lysine acetylation on the ε-amine group not only neutralizes the positive charge of the amine group, enhances the hydrophobicity, and increases the size of the lysine side chain, but also provides platforms for binding by proteins involved in chromatin and gene regulations. Histone acetylation/deacetylation is an essential process in the epigenetic regulation of diverse biological processes, including environmental stress responses in plants [1–3]. The level of histone acetylation is determined by the activity of both histone acetyl-transferases (HAT) and deacetylases (HDAC). Histone acetylation can be regulated by primary metabolism, as key metabolites such as acetyl-CoA and NAD+ are involved in protein acetylation and deacetylation processes, and their cellular levels may regulate the activity of HATs and HDACs [4, 5]. Plant metabolism is largely regulated to adapt to the fluctuating environment. Thus, the collaborative program between epigenetic dynamics and metabolism is further interconnected with environmental cues, which may optimize plant adaptive responses and growth [2].

Recently, it is shown that histones can be acylated with short-chain fatty acids, including propionylation, butyrylation, crotonylation, 2-hydroxyisobutyrylation, succinylation, malonylation, glutarylation, and β-hydroxybutyrylation in animal cells [6–8]. These modifications are similar to lysine acetylation but are distinct in hydrocarbon chain length and hydrophobicity or charge. Studies in animal cells suggest that histone lysine acylations affect gene expression and may be functionally different from histone lysine acetylation [9]. Histone lysine acylations are shown to be regulated by metabolism. For instance, intracellular crotonyl-CoA stimulates transcription through histone crotonylation [10]. In addition, β-hydroxybutyrylation is induced significantly during prolonged fasting in mouse liver and is associated with gene upregulation in starvation-responsive metabolism pathways [11].

It has been reported that histone acetyltransferases (HATs) and deacetylases (HDACs) could be involved in histone acylation dynamics [12]. For instance, mammalian CBP/p300, a histone acetyltransferase, catalyzes both histone acetyl and non-acetyl acylation [6, 10, 13, 14]. All seven human sirtuins (SIRT1-7) that were originally annotated as histone deacetylases based on sequence homology to the yeast sir2 histone deacetylase, have been described as having diverse deacylase activities, albeit weaker than their deacetylase activities [9, 13, 15]. Other studies have shown that Zn-finger domain-containing HDACs are also involved in decrotonylation of histone lysine residues [12].

Histone acylation has not been studied yet in plants. Being photosynthetic organisms, plants are autotrophic and there are significant differences in primary and energy metabolic and signaling pathways. For instance, glycolysis is induced under both biotic and abiotic stresses, and tricarboxylic acid (TCA) cycle is particularly high during light to dark transition [16, 17]. It remains unknown how histone acylation and acetylation interact to regulate gene expression in plants that have distinct regulatory pathways of primary metabolism.

In this work, we first analyzed rice histone butyrylation (Kbu) and crotonylation (Kcr) sites by mass spectrometry, then compared genome-wide distribution of Kbu and Kcr and lysine acetylation (H3K9ac) together with transcriptomic analysis in rice. To study whether altered metabolic activity affected histone acylations and acetylation, we treated rice plants by continuous darkness (starvation) and submergence and investigated genome-wide changes of Kbu, Kcr, and H3K9ac and gene expression. Finally, we tested histone deacylation activities of several rice HDACs. Our results indicate that Kbu and Kcr are robust histone modification marks that regulate gene expression in rice. Rice histone acylations and acetylation are dynamically regulated by environmental cues and are likely to be removed by different mechanisms.

## Results

### Identification of rice histone lysine butyrylation (Kbu) and crotonylation (Kcr) sites

**Fig. 1:**
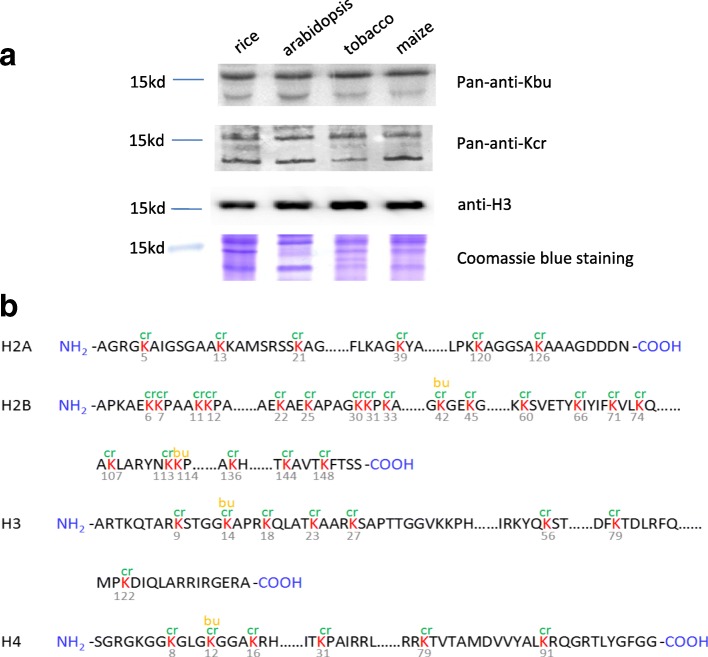
Identification of rice histone Kbu and Kcr modifications. **a** Detection by immunoblotting of Kbu and Kcr in histones isolated from rice, Arabidopsis, tobacco, and maize seedlings. **b** Rice histone Kbu and Kcr sites detected by mass spectrometry (see Additional file 1: Figure S1 and Additional file 2: Dataset 1)

To study whether histone lysine acylations exist in plants, we first performed immunoblotting with specific antibodies and detected histone lysine butyrylation (Kbu) and lysine crotonylation (Kcr) in Arabidopsis, rice, maize, and tobacco (Fig. 1a). In order to identify plant histone Kbu and Kcr sites, we used previously reported mass spectrometry methods to analyze histone proteins isolated from rice seedlings [8]. The analysis detected Kbu at N-terminal tails of H3 (H3K14bu) and H4 (H4K12bu), and at the core region of H2B (K42 and K134) of rice histones (Fig. 1b; Additional file 1: Figure S1; Additional file 2). H3K14bu exists in yeast but not detected in mammalian cells, whereas H4K12bu is detected in both yeast and mammalian cells [6]. By contrast, a large number of Kcr sites were detected in the N-terminal tails of H3 (K9, K14, K18, K23, and K27) and H4 (K8, K12, and K16) (Fig. 1b), most of which (K9cr, K18cr, K23cr, and K27cr of H3 and K8cr, K12cr, and K16cr of H4) were also identified in human and mouse cells [8]. Several sites of H2A and many sites of H2B were found to be crotonylated in rice (Fig. 1b). In total, 45 Kcr sites were identified in rice histones. Kcr sties were found not only in the N-terminal but also in the globular domain and C-terminal end of core histones.

### Comparative analysis of rice genome-wide histone Kbu, Kcr, and H3K9ac

**Fig. 2:**
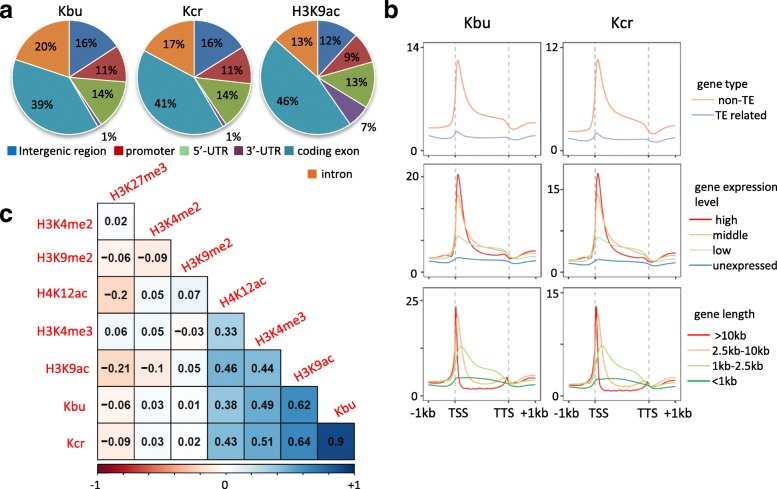
Distribution of histone Kbu and Kcr modifications in the rice genome. **a** Distribution of histone Kbu/Kcr peaks in different regions of the rice genome. **b** Average genome-wide occupancies of Kbu and Kcr in different categories of genes. **c** Correlogram of Kbu, Kcr, and other histone modifications. The genome was divided into 1-kb bins and TPM values of each bin were used to calculate correlation coefficients

In order to explore chromatin function of histone Kbu and Kcr in rice, we performed anti-Kbu and anti-Kcr chromatin immunoprecipitation-sequencing (ChIP-seq) assays of 12-day-old wild type seedlings. To study the relationship between histone acylation and histone acetylation, we also performed ChIP-seq with anti-H3K9ac antibody. More than 30,000 Kbu and Kcr peaks were identified (Additional file 1: Table S1), indicating that the two histone acylation modifications are widely distributed in the rice genome, although some reads/peaks may be associated with Kbu- or Kcr-modified chromatin proteins other than histones. The Kbu and Kcr modifications showed a similar genomic distribution and profile as H3K9ac in rice (Fig. 2a). The majority of Kbu and Kcr peaks were located in the genic regions, and the modifications were highly enriched at the transcription start site (TSS) of protein-coding genes and were positively related to gene expression (Fig. 2a, b). By contrast, histone Kbu and Kcr levels were very low in transposable element (TE)-related genes (Fig. 2b). Kbu and Kcr were highly correlated with each other and with the active histone marks H3K9ac and H3K4me3, but did not correlated with H3K9me2, H3K4me2, or H3K27me3 (Fig. 2c). The genomic and genic distributions of Kbu and Kcr were somewhat similar to those of H3K9ac in rice [18]. A positive correlation of Kbu and Kcr with gene length was also observed (Fig. 2b). Altogether, the analysis suggests that Kbu and Kcr are active chromatin marks in rice.

**Fig. 3:**
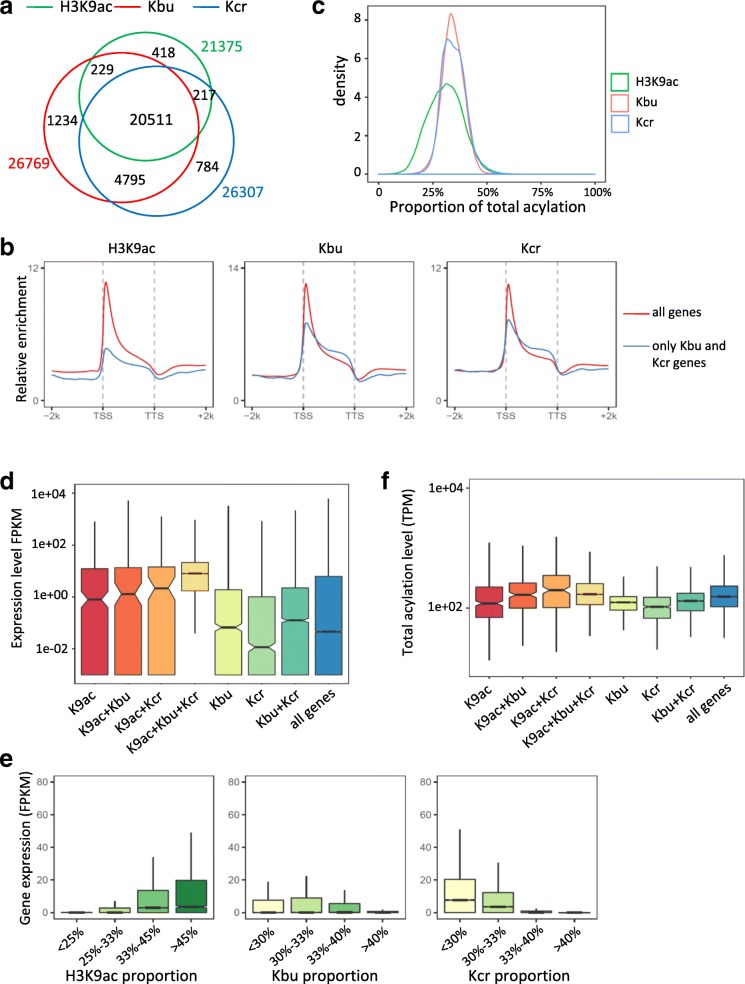
Different combinations of Kbu, Kcr, and H3K9ac modifications affect gene expression. **a** Venn diagram of Kbu/Kcr/H3K9ac modified genes. **b** Average genome-wide occupancies of H3K9ac, Kbu, and Kcr in all genes and genes with only Kbu and Kcr modifications (*N* = 4795). **c** Density curve of H3K9ac/Kbu/Kcr modification proportions in all genes. **d** Expression levels of genes with different modification profiles. Numbers of genes: H3K9ac (*N* = 418), H3K9ac + Kbu (*N* = 229), H3K9ac + Kcr (*N* = 217), H3K9ac + Kbu + Kcr (*N* = 20511), Kbu (*N* = 1234), Kcr (*N* = 784), Kbu + Kcr (*N* = 4795), all genes (*N* = 56384). *Y*-axis stands for gene expression levels calculated by log10 (FPKM + 0.001). **e** Expression levels of genes with different proportion of H3K9ac, Kbu, and Kcr under normal conditions. **f** Total acylation levels of genes with different combinations of the marks

From the two replicates, 26 769 Kbu- and 26 307 Kcr-marked genes were identified in rice, most of which (25 306) were co-marked by both modifications, indicating a high concurrence of the two marks (Fig. 3a). Interestingly, almost all (> 96%, 20,511 out of 21,375) H3K9ac-marked genes were also modified by Kbu and Kcr, while more than 5000 genes were marked by Kbu or Kcr, but not by H3K9ac (Fig. 3a). However, it is not excluded that these genes are marked by acetylation at other histone lysine residues. In these genes, Kbu and Kcr displayed relatively lower levels at TSS but higher levels in gene body (Fig. 3b). The ChIP-seq data were validated by ChIP-qPCR tests on modified and unmodified genes (Additional file 1: Figure S2). We analyzed quantitatively Kbu, Kcr, or H3K9ac proportion of the total acylation in the marked genes and found that H3K9ac portion varied in a wider range than that of Kbu or Kcr (Fig. 3c), suggesting that H3K9ac is more dynamic.

To study potential roles of histone acylations in gene expression, we performed RNA-seq analysis of the same plant materials (Additional file 1: Table S2). To investigate functional relationship between Kbu, Kcr, and H3K9ac in gene expression, we analyzed expression levels of genes modified by different combinations of the marks (Fig. 3d). Genes marked by H3K9ac showed higher expression than those marked only by Kbu or Kcr. Genes marked by a combination of two marks showed higher expression than the mono-marked genes. Genes with all three marks displayed the highest expression levels. In genes with the three marks, those with higher portion of H3K9ac relative to Kbu or Kcr displayed higher expression levels (Fig. 3e). However, the total acylation level of genes with the three marks was not higher than those with only two marks (Fig. 3f). The analysis suggests that H3K9ac is predominant in gene activity and that Kbu and Kcr may contribute to H3K9ac-regulated active chromatin state. Genes marked by Kbu or Kcr only (tags per million mapped tags per kb, TPM > 50) showed low expression levels (FPKM < 0.1) and were enriched in stress and abiotic stimulus responses. Many of them are highly induced under stress (Additional file 1: Figure S3), hypothesizing that Kbu and Kcr may play a role in poising genes for stimulus-induced activation.

### Kbu, Kcr, and H3K9ac changes in rice under starvation and submergence

The substrates for histone acetylation/acylation, such as acetyl-CoA, butyryl-CoA, and crotonyl-CoA, are important cellular primary metabolites. Stress conditions such as prolonged darkness (starvation) or submergence (switching to anaerobic respiration) affect greatly plant primary metabolism. To study whether starvation and submergence altered Kbu/Kcr/H3K9ac, we treated rice seedlings in darkness for 48 h or under submergence for 6 h. qRT-PCR analysis of starvation and submergence-induced marker genes confirmed the effectiveness of the treatments (Additional file 1: Figure S4A). The materials were harvested at 6 pm and used for ChIP-seq with anti-Kbu, anti-Kcr, and anti-H3K9ac and RNA-seq.

**Fig. 4:**
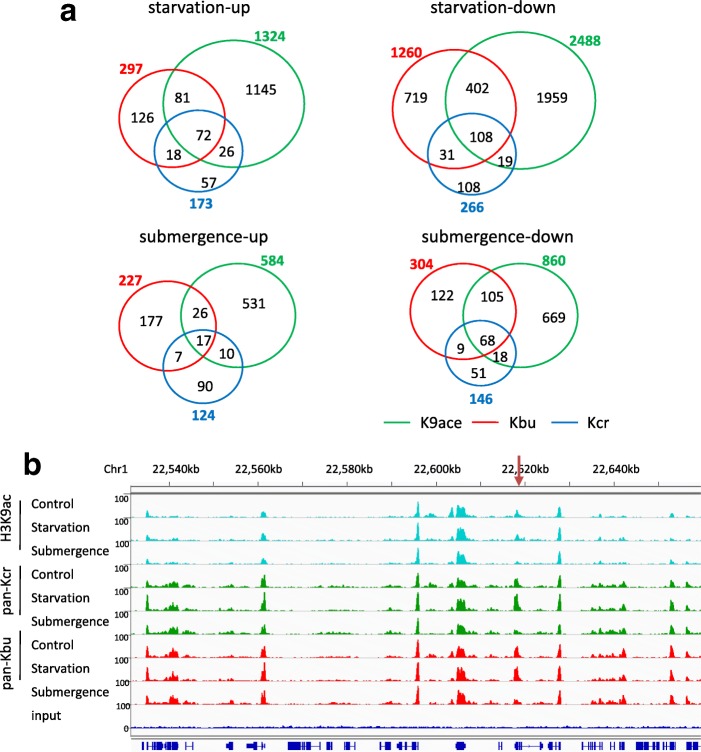
Kbu, Kcr, and H3K9ac changes in rice under starvation and submergence. **a** Overlapping of genes with Kbu/Kcr/H3K9ac changes (increase and decrease) after starvation and submergence treatments. Genes with TPM changes > 1.5-fold in both replicates were taken into consideration. **b** Igv screenshots showing H3K9ac, Kbu, and Kcr peaks in different treatments. Arrow indicates a differentially modified region (see Additional file 1: Figure S5)

ChIP-seq analysis revealed about similar numbers of modified genes between the treatments and the controls (Additional file 1: Table S1). Genes with > 1.5-fold changes of TPM in both replicates were defined as differentially modified. With these criteria, 1324 and 584 genes showed increases of H3K9ac, while 2488 and 860 genes showed decreases of the mark respectively under starvation and submergence (Fig. 4). Kbu levels were augmented in 279 and 277 genes, but reduced in 1260 and 304 genes under starvation and submergence, respectively. Relatively fewer genes showed Kcr changes after the treatments (Fig. 4a). Thus, H3K9ac appeared more dynamic than Kbu and Kcr. Starvation resulted in more changes of H3K9ac than submergence, suggesting that cellular energy levels might have a higher impact on histone acetylation in plants. Interestingly, more than 50% genes that showed Kcr changes after starvation (both increase and decrease) or submergence (decrease) also showed changes of Kbu, supporting the concurrency hypothesis of the two marks on many genes. By contrast, only about 10–22% genes with H3K9ac changes showed changes of Kbu or Kcr after starvation or submergence (Fig. 4a), suggesting that H3K9ac and Kbu/Kcr regulate different sets of genes in these conditions and that regulation of H3K9ac and Kbu/Kcr dynamics might involve different mechanisms. Still, 180 (72 up and 108 down) and 85 (17 up and 68 down) genes showed concurrent changes of the three marks under starvation and submergence, respectively (Fig. 4; Additional file 1: Figure S5).

In parallel, RNA-seq analysis revealed that starvation and submergence resulted in differential expression (FDR < 0.05, fold change > 4) of 1967 (1045 upregulated, 922 downregulated) and 1305 (487 upregulated, 818 downregulated) genes, respectively (Additional file 1: Table S2; Figure S4B). GO analysis indicated that the differentially expressed genes induced by starvation are enriched in energy metabolic processes and in stress and stimulus response pathways (Additional file 1: Table S3). Submergence-affected genes were enriched in nucleic acid metabolism and DNA-binding processes in addition to stress and stimulus response pathways (Additional file 1: Table S4). Some of the enriched gene categories were also enriched for H3K9ac, Kbu, and Kcr changes (Additional file 1: Table S3, Table S4). Remarkably, transcription factor genes were found to be enriched among those with increased or decreased H3K9ac under starvation and submergence and with decreased Kbu and Kcr under submergence (Additional file 1: Table S3, Table S4).

**Fig. 5:**
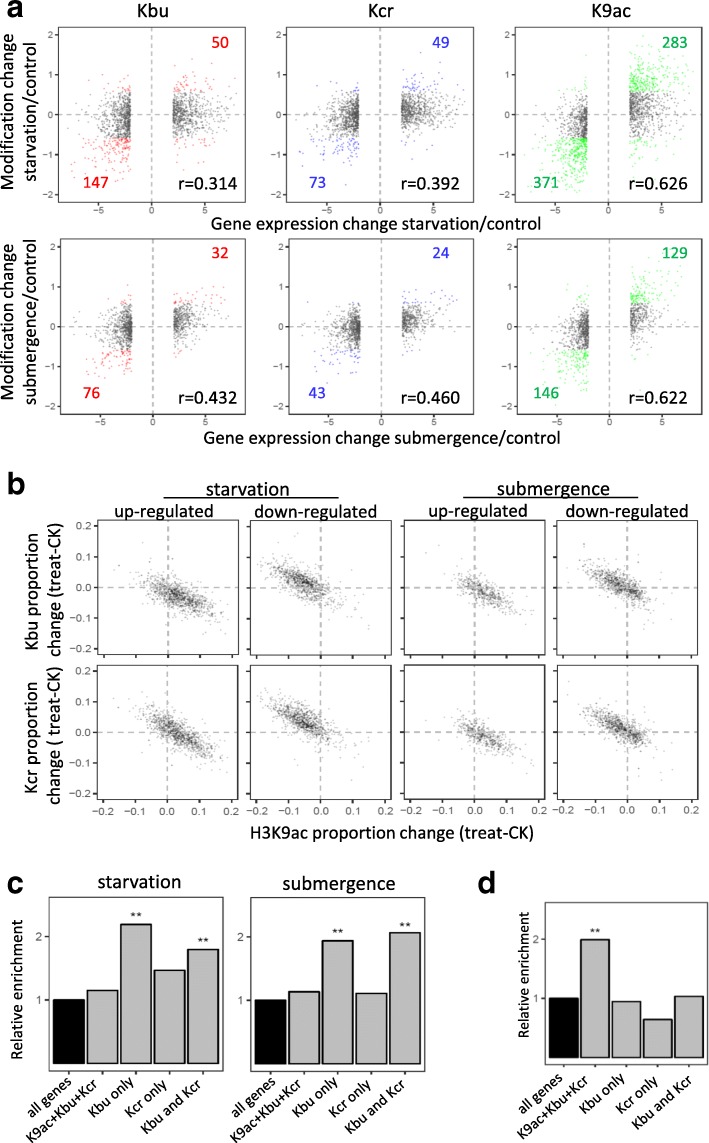
Role of Kbu, Kcr, and H3K9ac dynamics in stress-induced and diurnal gene expression. **a** Plots of Kbu, Kcr, and HK9ace changes of differentially expressed genes in rice under starvation and submergence. *X*-axis: gene expression fold change (log2); *Y*-axis: gene lysine acylation changes (log2); genes with acylation level (TPM) change > 1.5-fold are marked by colors. **b** Scatter plot showing the proportion change of H3K9ac and Kbu/Kcr in starvation/submergence DEGs. Proportion of each modification was calculated by its TPM in gene body divided by total acylation TPM value (K9ac + Kbu + Kcr) in each gene. **c** Enrichment of starvation and submergence upregulated genes with different combinations of the acylation marks. **Significant enrichments (*p* < 0.01). **d** Enrichment of circadian-regulated genes marked by different combinations of lysine acylations (see Additional file 1: Figure S6). **Significant enrichments (*p* < 0.01)

To investigate the relationship between histone acetylation/acylation dynamics and gene expression changes, we analyzed changes of H3K9ac, Kbu, and Kcr TPM levels of the differentially expressed genes induced by the stresses. The analysis indicated that H3K9ac change was positively correlated to gene expression change (*r* > 0.6), while the correlation between Kbu or Kcr and gene expression changes is weak (Fig. 5a). In fact, quantitative analysis of concurrent changes of Kbu, Kcr, and H3K9ac proportions revealed that H3K9ac portion increased while Kbu or Kcr portion decreased in upregulated DEGs, whereas the reversed trend was observed in the downregulated genes (Fig. 5b). This analysis supported the above hypothesis that H3K9ac was predominant in gene activation during stress responses. Starvation and submergence resulted in respectively 283 and 129 upregulated (> 4-folds) genes with elevated H3K9ac (> 1.5-folds), and 371 and 146 downregulated (> 4-folds) genes with reduced H3K9ac (> 1.5-folds) (Fig. 5a; Additional file 3). Under starvation, besides photosynthetic genes, glycolytic genes (such as F1.6BP and GAPDH) showed reduced expression and H3K9ac, whereas genes involved in energy mobilization such as those encoding lipases and glycosyl hydrolases displayed higher expression and H3K9ac (Additional file 3). Under submergence, the emblematic marker gene, Sub1C (Os09g11460, an AP2 transcription factor) [19]and gibberellin biosynthetic genes GA20oxidase were found to be among those with upregulation of expression and H3K9ac. Several ZIM (involved in jasmonate-signaling) and VQ domain genes, in addition to many other transcription factor genes, showed reduced expression and H3K9ac (Additional file 3). With the same criteria, relatively fewer genes showed concurrent changes of expression and Kbu or Kcr modification (Fig. 5a; Additional file 3). Most of the genes that displayed concurrent increases or decreases of the three marks, such as Sub1C and Os02g52650 (Fig. 4a; Additional file 1: Figure S4C), were respectively up- and downregulated (Additional file 4).

### Kbu may poise external stress-induced gene expression

To check whether Kbu and Kcr played a role to poise genes for stimulus-induced expression, we analyzed upregulated genes under starvation and submergence and found that the genes marked by Kbu alone or by Kbu and Kcr were significant enriched compared to the genome-wide averages (Fig. 5c). By contrast, no enrichment was observed for genes concurrently modified by the three marks or by Kcr alone. The analysis supported the hypothesis that Kbu might have a function to poise genes for environmental stress-induced activation.

To study whether Kbu had a function to poise gene activation by internal signals, we identified genes that displayed circadian expression in the rice seedlings at four diurnal time points by RNA-seq (Additional file 1: Table S2) and also analyzed H3K9ac by ChIP-seq of the samples (Additional file 1: Table S1). Genes with significant expression changes (FDR < 0.05, fold change > 2) in more than two of the six comparisons were considered to have diurnal rhythm (Additional file 1: Figure S6A). By these criteria, 2867 diurnal cycling genes (about 13% of expressed genes) were found, and their expression dynamics was tightly correlated with that of H3K9ac levels (Additional file 1: Figure S6B and S6C). Genes marked concurrently by the three marks were enriched for the diurnal expression, whereas those marked by Kbu and Kcr were not enriched (Fig. 5d). Thus, Kbu appeared to be preferentially involved in poising genes for stress-induced activation, whereas H3K9ac is associated with internal circadian gene expression.

### SRT2 is involved in erasure of histone Kcr in rice

**Fig. 6:**
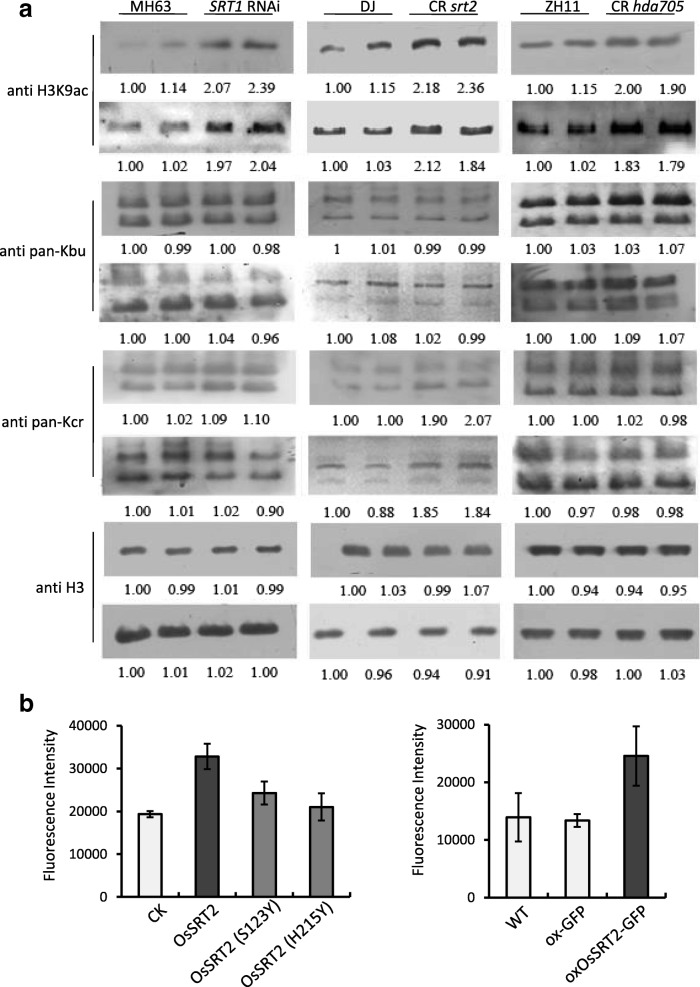
Rice SRT2 has a decrotonylase activity. **a** Histone H3K9ac, Kbu, and Kcr levels in *SRT1* RNAi, *srt2* CRISPR, and *hda*705 CRISPR plants compared to wild types (MH63, DJ, and ZH11) were detected by immunoblotting. Each sample was loaded twice and two replicates for each immunoblot are shown. Relative quantified signals of each band are indicated with the first wild type loading set as 1. **b** In vitro lysine decrotonylation activity of OsSRT2 by fluorometric assays. Left panel, assays with *E. coli*-produced OsSRT1-GST protein. GST and mutated OsSRT2 (S123 Y and H215 Y in the catalytic domain) were used as control. Right panel: assays with OsSRT1 protein produced in transient transfected tobacco cells. Bars are means ± SD from three biological replicates

Mammalian sirtuin and Zn-finger HDAC proteins are found to have debutyrylase and/or decrotonylase activities [9, 20]. To investigate whether rice sirtuin proteins could regulate histone Kbu and Kcr, we analyzed *OsSRT1*RNAi plants [21, 22] and *ossrt2* CRISPR mutants (Additional file 1: Figure S7) by immunoblots using anti-Kbu and anti-Kcr antibodies (Additional file 1: Figure S8; Fig. 6a). In *OsSRT1* RNAi plants, we did not detect any clear difference of Kbu or Kcr but a clear increase of H3K9ac compared with wild type plants. In *ossrt2* mutants, an increase of both H3K9ac and Kcr, but not Kbu, was observed. In addition, we analyzed a Zn-binding RPD3 type HDAC HDA705 [23, 24]. Its mutation resulted in some increase of H3K9ac but had no clear effect on Kbu and Kcr. The results suggest that OsSRT2 has a decrotonylase activity. The in vitro decrotonylase activity of *Escherichia coli*-produced OsSRT2-GST fusion protein was confirmed by fluorometric assays (Fig. 6b). Substitutions of two conserved residues in the catalytic domain of SIR2 proteins in SRT2 abolished the activity (Fig. 6b). The relatively weak effect of *ossrt2* mutation and no effect of *OsSRT11* RNAi on Kbu or Kcr might suggest a redundant function of OsSRT1 and OsSRT2 in lysine deacylation in rice.

## Discussion

Lysine butyrylation (Kbu) and crotonylation (Kcr) result in extended hydrocarbon chains that increase the hydrophobicity and bulk of the modified lysine residues in histones exceeding that of lysine acetylation. Whether and how these epigenetic modifications affect gene expression and how they are regulated remains unknown in plants. In this work, we identified a large number of rice histone Kbu and Kcr sites in rice. Although many Kbu and Kcr sites are conserved in mammalian histones, several ones appear to be specific in plants. For instance, H3K14bu seems to exist only in plant and yeast. In addition, we showed that rice Kbu and Kcr are peaked at the TSS regions which is different from human or mouse histone Kbu and Kcr that have also a peak in enhancers at the 5′ region in addition to the TSS [8]. This may be due to architectural difference of cis-regulatory elements between plant and mammalian genes. We showed that histone Kbu and Kcr mark a large number of genes in rice. The majority of Kbu- and Kcr-marked genes are also marked by H3K9ac and are transcriptionally active, which seems to be distinct from results obtained in mammalian cells, in which it has been shown that histone acetylation dynamically competes with butyrylation of highly active gene promoters. The high degree of overlapping between H3K9ac and Kbu/Kcr in the rice genome supports the model in which genomic loci are distinguished not by the presence or absence of a particular acylation but probably by the proportional mixture of distinct acylations. The differential changes of H3K9ac and Kbu/Kcr induced by starvation and submergence suggest that environmental and metabolic cues control proportion of histone acylations in plants, which controls the expression of responsive genes. Several recent studies in animal cells have demonstrated that alterations in the proportional mixture of histone acylations have functional consequences and correlate with distinct physiological states [10, 11, 25–27]. Higher correlations between H3K9ac and expression changes compared to Kbu and Kcr observed in this study suggest that histone acylations may provide a platform for H3K9ac during gene activation in plants. In line with this hypothesis is data showing that Kbu may poise for stress-induced gene activation. The observations that starvation and submergence induced changes of H3K9ac and Kbu/Kcr from different sets of genes and that relatively few genes showed concurrent changes between expression and Kbu or Kcr suggest that the two histone acylation marks and H3K9ac have non-redundant functions in these contexts. Therefore, relative levels of histone acylation and acetylation regulated by environmental and metabolic changes may be a mechanism that fine-tunes regulation of gene expression for plant adaptation.

It has been shown that histone acetyltransferases, such as CBP/p300, catalyze both histone acetyl and non-acetyl acylation [6, 10, 13, 14] and that the different acyl-CoAs compete for binding to the enzyme [9]. Acetyl-CoA produced from cytosolic/nucleic citrate by ATP citrate lyase (ACL) is the major source for histone acetylation. Depletion of acetyl-CoA by knockdown of *ACL* reduces histone lysine acetylation in both animal and plant cells [28, 29]. Reduction of the cytoplasmic and nuclear pools of acetyl-CoA led to an increase in CBP/p300-catalyzed Kcr in animal cells and the increase in Kcr could be reduced by replenishing the acetyl-CoA pools [10]. Thus, under conditions in which acetyl-CoA is reduced, other acyl-CoA forms will be used more often to modify histones. The relative stability of Kbu and Kcr in rice under starvation and submergence may be due to the possibility that under these conditions tricarboxylic acid cycle (TCA) activity is low resulting in lower cytosolic/nuclear acetyl-CoA, which may be in favor of stabilizing histone Kbu/Kcr acylation. Furthermore, the differential dynamism between H3K9ac and Kbu/Kcr during starvation and submergence may be also related to difference in their erasure mechanism, as only the mutation of *ossrt2* had an effect on Kcr, while the three tested HDAC (SRT1, SRT2, and HDA705) all resulted in increases of H3K9ac (Fig. 6). This is in line with the suggestion that non-acetyl histone acylation is mainly removed by sirtuin HDACs, while lysine acetylation can be reversed by both sirtuin and Zn-containing HDAC [9], although mammalian HDAC1 is shown to have a decrotonylase activity [12] . However, it is not excluded that anti-Kbu and anti-Kcr antibodies that detect the modifications at all histone sites may not readily detect changes at specific histone lysine sites. A mixture of signals (Kcr or Kbu on different lysines) may change less compared to a single modification (H3K9ac). In addition, Kbu/Kcr/H3K9ac may be regulated at different time points during the stresses. It is also not excluded other factors related to stresses may be involved in the differential changes of histone acylations.

## Conclusions

We identified many plant histone Kbu and Kcr sites, which mark a large number of genes in rice. Kbu and Kcr sites are enriched at 5′ region of active genes and most of the H3K9ac-marked genes are co-marked by Kbu and Kcr. However, histone Kbu, Kcr, and H3K9ac display different dynamics in responding to environmental and metabolic cues and have non-redundant functions in gene activation in rice under starvation and submergence. Histone acylation (i.e., Kbu) are likely to poise genes for stress induction and provide a platform for H3K9ac during gene activation in plant. Our data indicate that the proportion of rice histone lysine acetylation and acylation is dynamically regulated by environmental and metabolic signals, which may serve as a mechanism to fine-tune epigenetic control of plant adaptation to environmental variation.

## Methods

### Plant material and treatments

Rice (*Oryza sativa*) wild type and mutant or RNAi plants were germinated and grown on hormone-free, one-half-strength Murashige and Skoog medium under 16/8 h of light/dark at 30 °C/25 °C. For starvation treatment, 10-day-old wild type DongJin (DJ) seedlings were moved into dark at 6:00 pm and cultured for 48 h. For submergence treatment, 12-day-old wild type seedlings were submerged in distilled water at noon (12:00 am) for 6 h. Seedling leaves of all samples were harvested at 6:00 pm for chromatin and total RNA extraction.

### Histone extraction and immunoblot analysis

Histone-enriched fractions were extracted from seedlings of Arabidopsis, rice, maize, and tobacco plants using EpiQuik Total Histone Extraction Kit (Epigentek USA, OP-0006-100). The histone-enriched fractions were used for immunoblot analysis; antibodies used in this study were anti-H3 (ab1791; Abcam), anti-H3K9ace (07-352; Millipore), anti-butyryllysine (PTM-301; PTM Biolabs), and anti-crotonyllysine (PTM-501; PTM Biolabs). Immunoblotting results were quantified using ImageJ (v1.6.0_24).

### Vector construction for the CRISPR/Cas9 system

The single-guide RNA (sgRNA) design for the CRISPR/Cas9 system and plasmid construction was conducted as previously described [30]. A web application tool, CRISPR-P (http://cbi.hzau.edu.cn/crispr), was used to select sgRNAs targeting exons of the target genes. The Cas9 destination vector was driven by the maize ubiquitin promoter for expression in rice, and sgRNA expression was driven by the pol III type promoter of U3 sgRNA. Gibson Assembly Cloning method [31] was used to mobilize sgRNA, after which the binary T-DNA vectors for co-expression of Cas9 and sgRNA were transformed into rice calli. Genomic sequences of *OsSRT2* (LOC_Os12g07950) and *OsHDA705* (LOC_Os08g25570) were obtained from Rice Genome Annotation Project (http://rice.plantbiology.msu.edu/). Mutation sites of *OsSRT2* and *OsHDA705* are shown in Additional file 1: Figure S7.

### LC–MS/MS and data analysis

Rice seedlings were grinded by liquid nitrogen; core histones were extracted by H2SO4 and digested by trypsin. Lysine crotonylation (Kcr) and butyrylation (Kbu) peptides were enriched by pre-washed antibody beads (PTM Biolabs, Hangzhou). The eluted peptides were cleaned with C18 ZipTips (Millipore) according to the manufacturer’s instructions, followed by analysis with LC–MS/MS. The resulting MS/MS data was processed using MaxQuant with integrated Andromeda search engine (v.1.5.2.8). Tandem mass spectra were searched against UniProt_*Oryza sativa* database (https://www.uniprot.org/) concatenated with reverse decoy database. False discovery rate thresholds for protein, peptide, and modification site were specified as 1%; minimum peptide length was set at 7; and the site localization probability was set as > 0.75.

### Chromatin immunoprecipitation

Two grams of rice seedling leaves was cross-linked by 1% formaldehyde and used for chromatin extraction. After sonication, chromatin fragments were incubated with antibody (anti-H3K9ac, anti-Kbu, and anti-Kcr)-coated beads (Invitrogen/Life Technologies; 10001D) overnight. Specificity of anti-Kbu and anti-Kcr was tested by dot blots (Additional file 1: Figure S8). After extensive washing, immunoprecipitated chromatin was de-cross-linked and retrieved for qPCR or sequencing. Anti-H3K9ace (07-352; Millipore), anti-butyryllysine (PTM-301; PTM Biolabs), and anti-crotonyllysine (PTM-501; PTM Biolabs) antibodies were used.

### ChIP-seq and data analysis

DNA from chromatin immunoprecipitation was used to construct sequencing libraries following the protocol provided by the Illumina TruSeqChIP Sample Prep Set A and sequenced on Illumina HiSeq 2000 with PE 150 method.

Trimmomatic (version 0.32) was used to filter out low-quality reads. Clean reads were mapped to the rice genome (MSU7.0) by Bowtie2 (version 2.2.5), allowing up to two mismatches. Samtools (version 0.1.19) was used to remove potential PCR duplicates, and MACS software (version 1.4.2) [32] was used to call histone modification peaks by default parameters (bandwidth, 300 bp; model fold, 10, 30; *p* value, 1.00e−5). Wig files produced by MACS software were used for data visualization by IGV (version 2.3.88). Genes that have modification peaks in gene body or 2 kb upstream in both replicates were defined as modified genes. Tags per kilobase of gene length per million mapped reads (TPM) was used in analyzing gene modification level; genes with TPM fold change > 1.5 in both replicates were considered as differentially modified genes.

In analyzing the relationship between different histone modifications, the rice genome were divided into 1-kb bins and the TPM values in each bin were used to calculate correlation coefficient; data were visualized by R package corrplot (v0.77). In drawing curves of modification distribution over genes, gene body and ± 2-kb regions were divided into 50-bp bins and the average TPM in each bin were plotted. Proportion of each modification was calculated by TPM values in gene body divided by the total acylation TPM value (K9ac + Kbu + Kcr) in each gene.

### RT-qPCR and ChIP-qPCR analysis

Total RNA was isolated using TRIzol reagent (Invitrogen). Four micrograms of total RNA was used to synthesize complementary DNA with reverse transcription kit (Promega). Real-time PCR was performed using SYBR Premix ExTaq (TaKaRa) on ABI 7500 real-time PCR system. Levels of rice *ACTIN* or input (chromatin sample without immunoprecipitation) were used for normalization in RT-qPCR or ChIP-qPCR, respectively. Primers used in RT-qPCR and ChIP-qPCR are listed in Additional file 1: Table S5.

### RNA-seq and data analysis

RNA samples were isolated using TRIzol reagent (Invitrogen). The RNA-seq libraries were prepared using the Illumina TruSeq RNA Sample Preparation Kit and sequenced on Illumina HiSeq 2000 with PE150 method.

RNA-seq data were filtered by Trimmomatic (version 0.33) to remove contaminations and low-quality reads. Tophat (version 2.0.14) and Cufflinks (version 2.2.1) [33] were used to map clean reads to rice genome (MSU 7.0) and calculate differentially expressed genes. Genes with adjusted *p* value < 0.05 and fold change > 4 in starvation or submergence treatments were considered differentially expressed.

### In vitro lysine decrotonylation assays

For OsSRT2 point mutations, forward and reverse primers covering the mutation sites (S123Y and H215Y) were designed (Additional file 1: Table S6) and the fusion PCR was adopted to obtain the mutated OsSRT2 coding sequences, which were then ligated to the protein expression vector PGEX4T-1. OsSRT2-GST, mutants of OsSRT2-GST, and GST proteins were expressed in *E. coli* and purified using Glutathione Sepharose 4B beads (GE Healthcare 17-0756101). The OsSRT2 full-length cDNA was cloned into pCambia 1301 vector between the 35S promoter and GFP sequences. The resulting vector 35S-SRT2-GFP and empty vector (35S-GFP) were transfected into tobacco leaf cells. After 36 h infection, the leaves were harvested. About 2-g samples were ground into fine powder in liquid nitrogen. Nuclear proteins were extracted as described previously [21] and suspended in 500 μL reaction buffer.

Reactions were performed as described [8] in a final volume of 200 μL per well in a 96-well microplate. Briefly, 1 μL of Boc-Lys(crotonyl)-AMC stocking solution (10 mM) and 50 μg purified protein or 80 μL nuclear lysis were added to the reaction buffer (25 mM Tris-HCl, 130 mM NaCl, 3.0 mM KCl, 1 mM MgCl2, 0.1% PEG8000, 500 μM NAD^+^, pH = 8.0) and incubated at 30 °C for 3 h. The reaction was then stopped by adding 25 μL of trypsin solution (25 mM Tris-HCl, 130 mM NaCl, 3.0 mM KCl, 1 mM MgCl, 30% isopropanol, 0.01 mg/mL trypsin, pH = 8.0). The resulting solvent was mixed and incubated at 30 °C for another 1 h. The fluorescence was analyzed by a fluorescence plate reader with excitation and emission wavelengths at 355 nm and 460 nm, respectively. Boc-Lys(crotonyl)-AMC were gifted by Prof. Yingming Zhao’s lab.

### Gene ontology analysis

Gene ontology classification provided in Rice Genome Annotation Project (http://rice.plantbiology.msu.edu/) was used to assign genes to a hierarchical biological process following the criteria of the Gene Ontology Consortium databases (http://www.geneontology.org/external2go/tigr2go). The *p* value of a particular GO item was calculated with Pearson’s chi-squared test and corrected by FDR, with an FDR cutoff of 0.05 as the significance threshold.

## Additional files

Additional file 1: **Figure S1.**-**Figure S8.** and **Table S1.**-**Table S6.** (PDF 469 kb)

Additional file 2: LC-MS results. (XLSX 76 kb)

Additional file 3: Lists of up- or downregulated genes with correlating changes of H3K9ac, Kbu, or Kcr under submergence or starvation. (XLSX 79 kb)

Additional file 4: Expression levels of genes with concurrent changes of Kbu, Kcr, and H3K9ac. (XLSX 40 kb)

Additional file 5: Review history. (DOCX 37 kb)
