# Supplementary material for: Dynamics and functional interplay of histone lysine butyrylation, crotonylation, and acetylation in rice under starvation and submergence
Source: Genome Biol. 2018 Sep 25;19:144. doi: 10.1186/s13059-018-1533-y (PMC6154804; doi:10.1186/s13059-018-1533-y)
Supplement: Supplementary file 1 — Figure S1.-Figure S8. and Table S1.-Table S6. (PDF 469 kb) [file 13059_2018_1533_MOESM1_ESM.pdf]

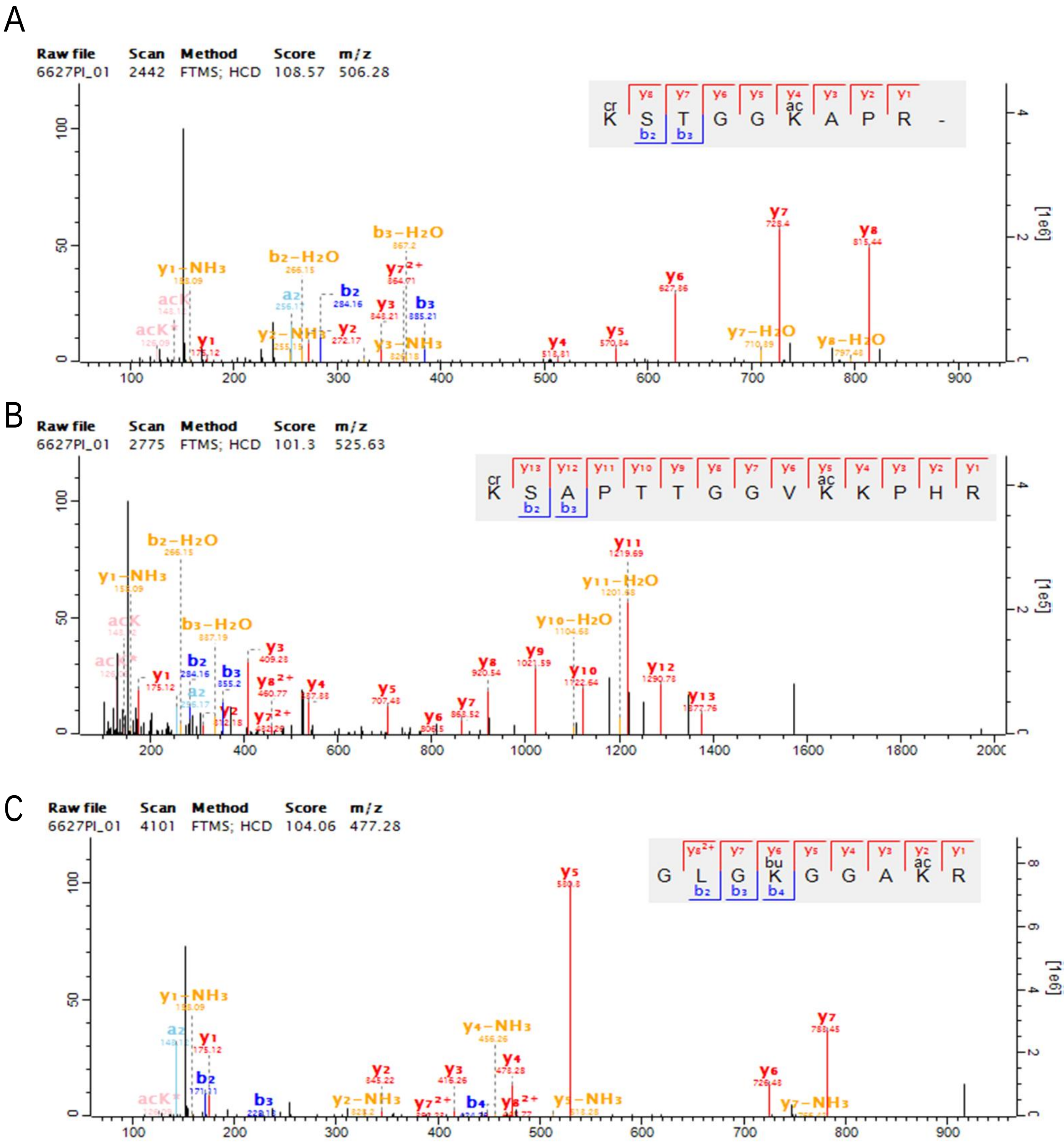

Figure S1. MS spectra of histone Kbu and Kcr sites in rice.

(A) MS/MS spectra of a tryptic peptide on histone H3K9 crotonylated peptide _K(cr)STGGK(ac)APR;

(B) MS/MS spectra of a tryptic peptide on histone H3K27 crotonylated peptide _K(cr)SAPTTGGVK(ac)KPHR;

(C) MS/MS spectra of a tryptic peptide on histone H4K12 butyrylated peptide _GLGK(bu)GGAK(ac)R.

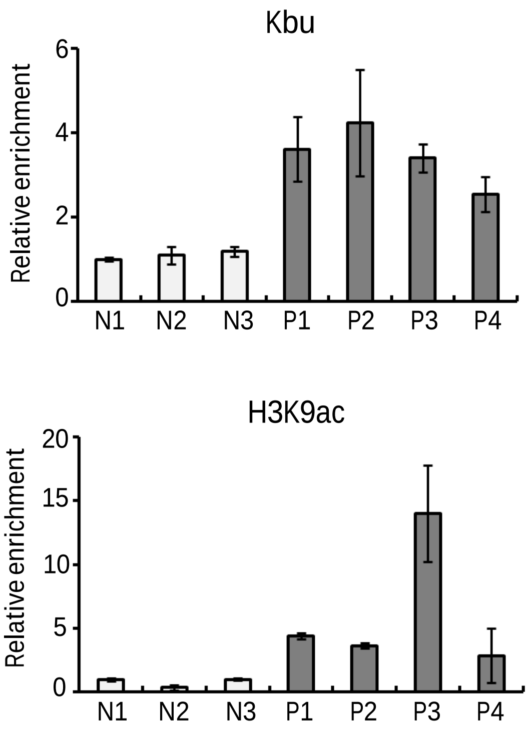

Figure S2. ChIP-qPCR validation of some modified genes (P1-P4) or unmodified genes (N1-N3). Bars are means ± SD from three biological replicates. Tested locus N1: LOC_Os12g37419; N2: LOC_Os01g66080; N3: LOC_Os01g65580; P1: LOC_Os09g11460; P2: LOC_Os07g47670; P3: LOC_Os02g52650; P4: LOC_Os01g66300.

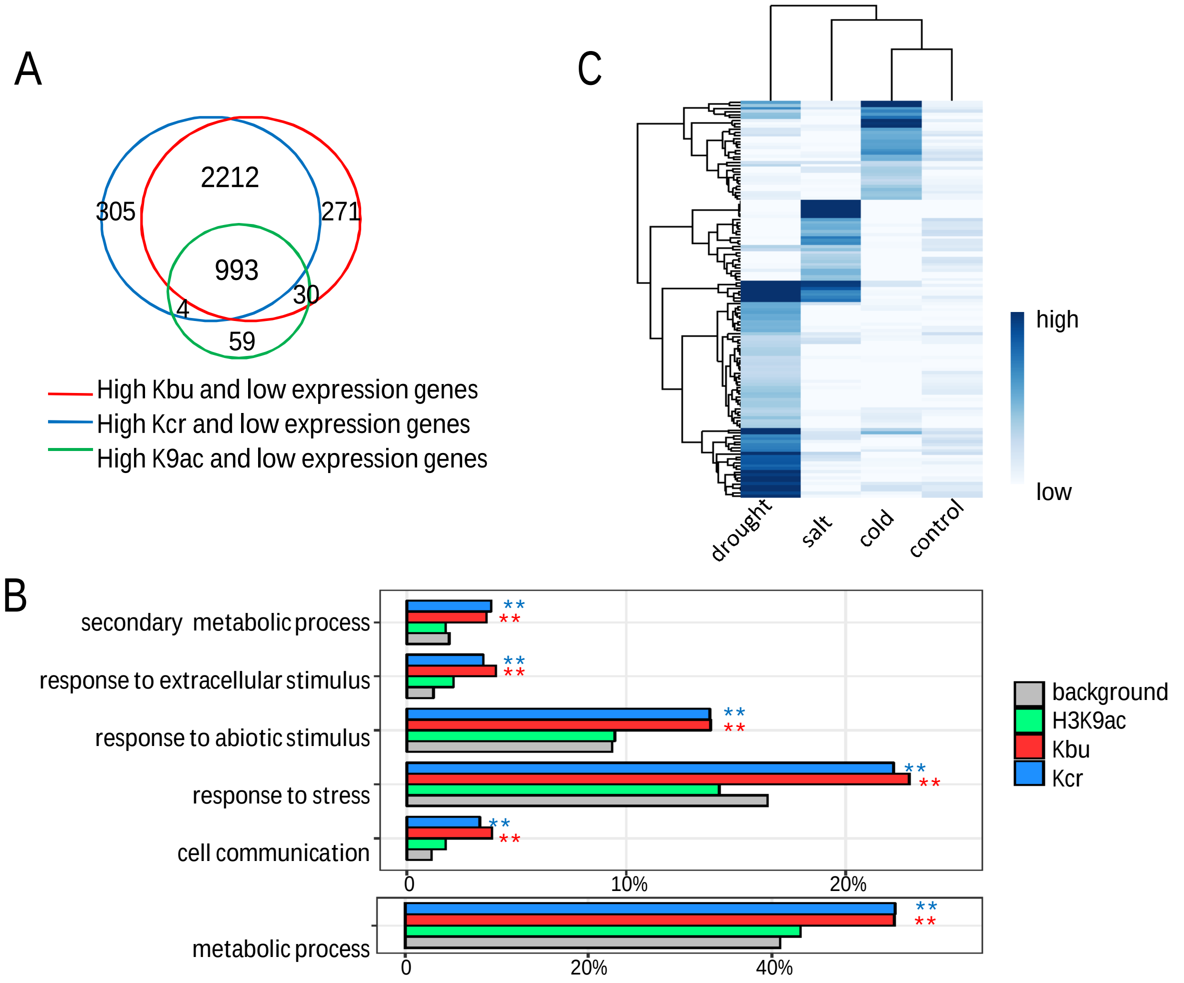

Figure S3. Kbu/Kcr-marked silent genes are enriched in stress responses and metabolic processes.

(A) Venn diagram of genes with very low expression (RPKM<0.1) and high modification levels (TPM in gene body >50) of H3K9ac (N=1120), Kbu (N=3506), and Kcr (N=3514).

(B) Gene ontology enrichment of H3K9ac-marked silent genes (N=1086) and Kbu/Kcr-specifically marked silent genes (N=2483 for Kbu, N=2517 for Kcr). Each group of genes was compared to all genes (background). X-axis: percentage of genes involved in the processes. ** FDR<0.01.

(C) Expression profile of some Kbu and Kcr-marked silent genes that are induced by stress treatments. Data of drought (GSE81462), salt (GSE101734), and cold (GSE67373) treatments were downloaded from GEO.

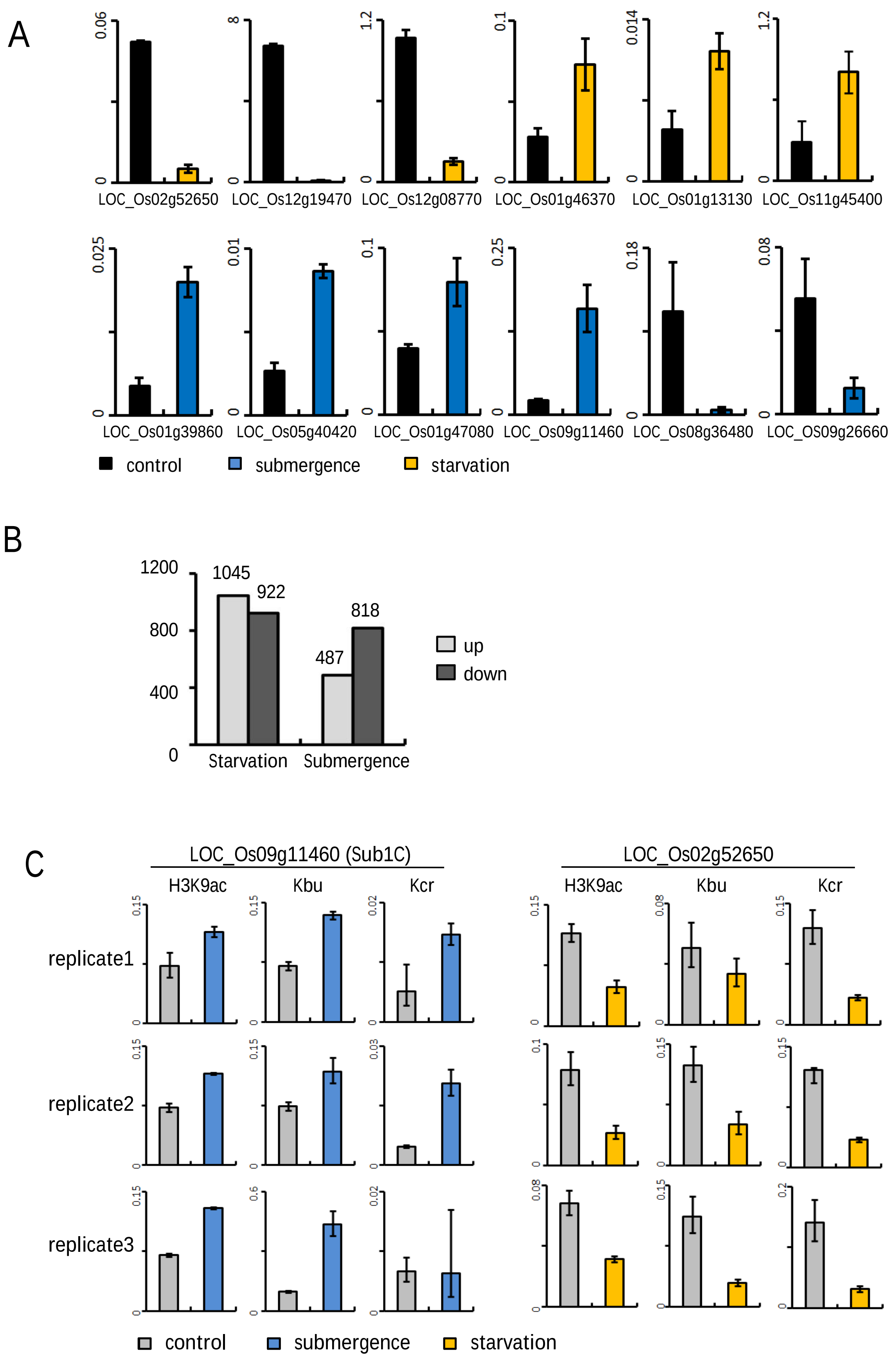
A
0.06
8
1.2
0.1
0.014
1.2
LOC_Os02g52650
LOC_Os12g19470
LOC_Os12g08770
LOC_Os01g46370
LOC_Os01g13130
LOC_Os11g45400
0.025
0.01
0.1
0.25
0.18
0.08
LOC_Os01g39860
LOC_Os05g40420
LOC_Os01g47080
LOC_Os09g11460
LOC_Os08g36480
LOC_OS09g26660
control
submergence
starvation
B
1200
800
400
0
1045
922
487
818
Starvation
Submergence
up
down
C
LOC_Os09g11460 (Sub1C)
LOC_Os02g52650
H3K9ac
Kbu
Kcr
replicate1
replicate2
replicate3
control
submergence
starvation

Figure S4. Validation of differentially expressed genes and their acetylation and acylation levels.

A. Validation of marker genes induced by starvation and submergence treatments. Y-axis stands for relative expression level compared with *Actin*. Bars are means ± standard deviation (SD) from three biological replicates. See also Table S3 for gene annotations.

B. Number of differentially expressed genes in starvation and submergence detected in the two replicates of RNA-seq (FDR<0.05, fold change>4).

C. ChIP-qPCR validation of H3K9ac, Kbu, and Kcr changes of two marker genes in the treatments. Y-axis stands for relative enrichment of corresponding modification, calculated by 0.5^Ct (IP–input). Bars are means ± SD from three technical replicates; three biological replicates are shown separately.

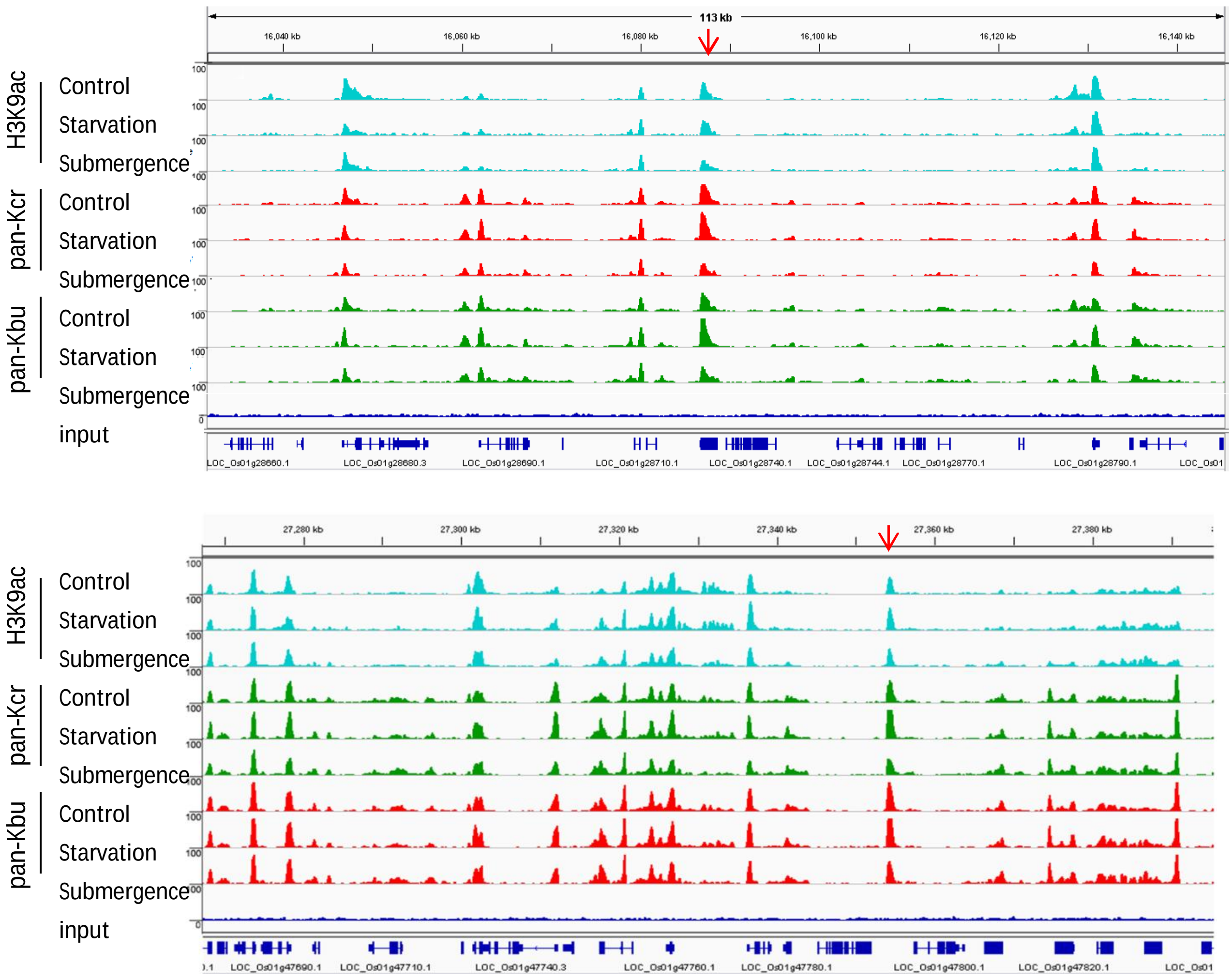

Figure S5. Igv screenshots showing K9ac, Kbu, and Kcr peaks in different treatments. Arrows indicate differentially modified regions.

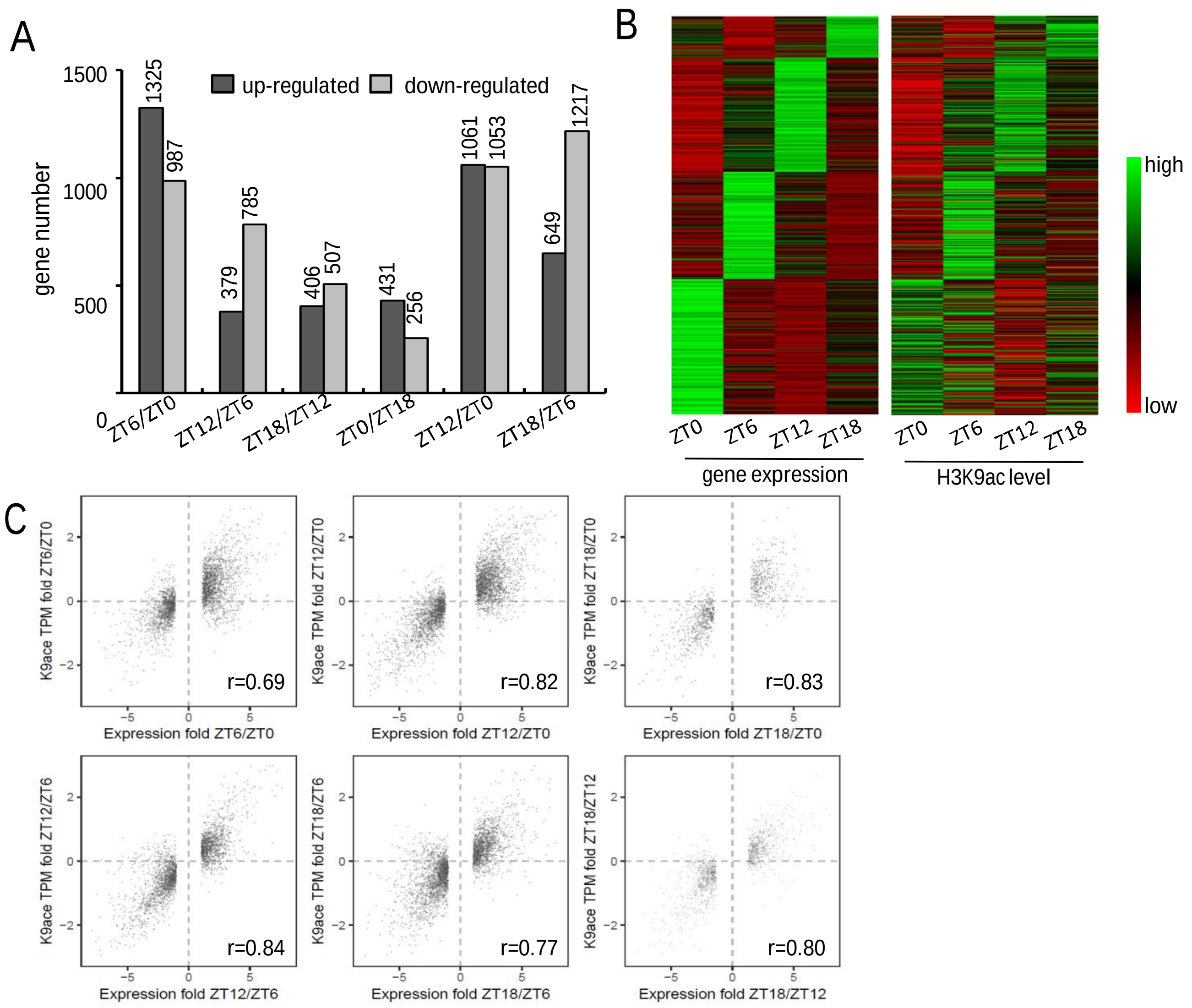

Figure S6. H3K9ac is tightly associated with circadian gene expression in rice

(A) Numbers of differentially expressed genes between 4 diurnal time points.

(B) Heatmaps of expression and H3K9ac modification profiles of 2781 cycling genes.

(C) H3K9ac change is positively related to gene expression change in diurnal rhythm. Expression fold changes and modification fold changes of differentially expressed genes in each comparison were plotted.

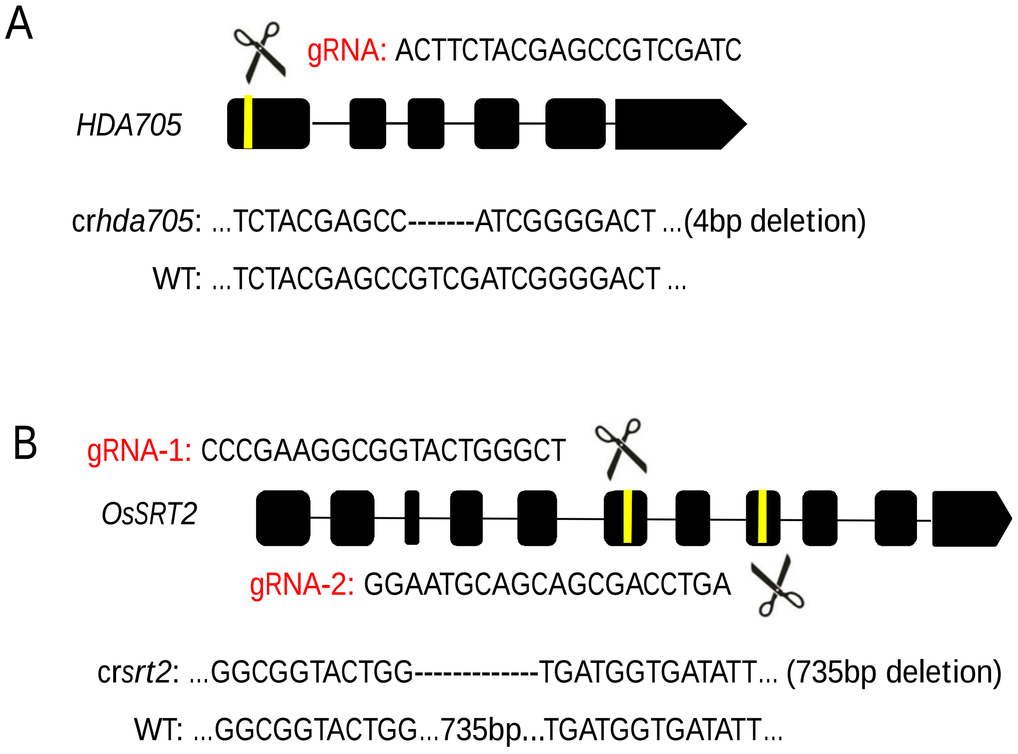

Figure S7. Construction of CRISPR mutants.
(A) Gene structure, gRNA, and mutation sequence of cr*hda705*;
(B) Gene structure, gRNA, and mutation sequence of cr*srt2*.

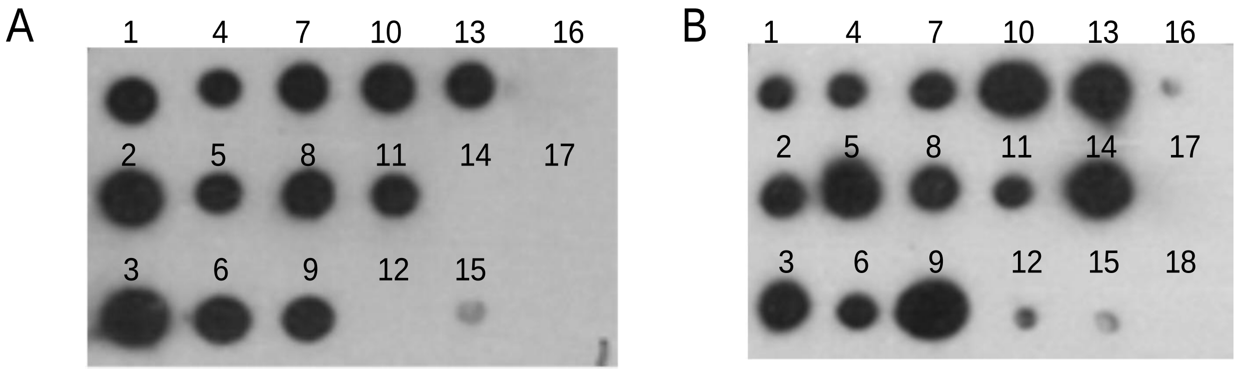

Figure S8. Specificity of pan anti-Kbu and pan anti-Kcr antibodies demonstrated by dot-blot assay

A. Dot-blot of pan anti-Kbu antibody. Peptide samples are as follows. 1: Kbu library 1ng; 2: Kbu library 4ng; 3: Kbu library 16ng; 4: Kbu peptide; 5: H4,butyryl-K31; 6: H4,butyryl-K79; 7: H3, butyryl-K79; 8: H3, butyryl-K122; 9: H2A, butyryl-K5; 10: H2B, butyryl-K11; 11: H2B, butyryl-K23; 12: H2B, acetyl-K23; 13: H2A, butyryl=K95; 14: Kac library; 15: propionyllysine (Kpr) library; 16: Kcr library; 17: K library control.

B. Dot-blot of pan anti-Kcr antibody. Peptide samples are as follows. 1: Kcr library 1ng; 2: Kcr library 4ng; 3: Kcr library 16ng; 4: Kcr peptide; 5: H2B crotonyl-K5; 6: H3, crotonyl-K79; 7: H4, crotonyl-K91; 8: H3, crotonyl-K122; 9: H2A, crotonyl-K125; 10: H3, crotonyl-K36; 11: H2B, crotonyl-K23; 12: H2a, crotonyl-K95; 13: H2B, crotonyl-K108; 14: pan crotonyl-H4; 15: Kac library; 16: propionyllysine (Kpr) library; 17: Kbu library; 18: K library control.

Table S1. ChIP-seq alignment summary

Sample	Antibody	Replicate number	Total reads (millions)	Uniquely aligned reads(millions)	Total aligned reads(millions)	Total peaks	Modified genes	R-squared TPM (all genes)
Control	Kbu	1	21.8	12.3 (56.4%)	20.7 (95.0%)	30,093	27,676	0.903
Control	Kbu	2	20.0	10.9 (54.5%)	18.6 (93.0%)	30,852	28,077	
Starvation	Kbu	1	19.7	10.7 (54.3%)	18.4 (93.4%)	28,337	26,007	0.936
Starvation	Kbu	2	21.7	11.6 (53.5%)	20.2 (93.1%)	28,859	26,471	
Submergence	Kbu	1	16.4	9.3 (56.7%)	15.7 (95.7%)	30,010	28,899	0.890
Submergence	Kbu	2	16.8	9.5 (56.5%)	16.1 (95.8%)	32,929	28,899	
Control	Kcr	1	19.2	10.9 (56.8%)	18.4 (95.8%)	29,792	27,027	0.872
Control	Kcr	2	20.8	11.6 (55.8%)	19.9 (95.7%)	32,235	28,892	
Starvation	Kcr	1	20.1	11.1 (55.2%)	18.9 (94.0%)	30,266	27,443	0.879
Starvation	Kcr	2	24.1	13.5 (56.0%)	23.2 (96.3%)	29,914	27,192	
Submergence	Kcr	1	20.5	11.5 (56.1%)	19.6 (95.6%)	33,265	28,981	0.856
Submergence	Kcr	2	18.2	10.3 (56.6%)	17.3 (95.1%)	33,251	29,052	
Control	H3K9ac	1	20.7	9.2(44.3%)	17.0(82.0%)	22,390	21,393	0.908
Control	H3K9ac	2	28.3	13.4(47.2%)	24.1(85.3%)	22,522	21,485	
Starvation	H3K9ac	1	19.4	6.7(34.7%)	13.6(69.9%)	21,562	20,338	0.906
Starvation	H3K9ac	2	26.8	10.3(38.5%)	20.1(75.1%)	21,829	20,515	
Submergence	H3K9ac	1	18.4	8.0(43.7%)	15.1(82.3%)	22,401	21,374	0.903
Submergence	H3K9ac	2	31.8	14.8(46.4%)	27.0(85.0%)	23,424	22,188	

Table S1. ChIP-seq alignment summary (continue)

sample	antibody	Replicate number	Total reads (millions)	Uniquely aligned reads(millions)	Total aligned reads(millions)	Total peaks	Modified genes	R-squared TPM (all genes)
ZT0	H3K9ac	1	29.6	17.8 (60.1%)	27.5 (92.9%)	21,916	20,598	0.8566
ZT0	H3K9ac	2	37.8	20.2 (53.4%)	33.8 (89.4%)	23,014	21,381	
ZT6	H3K9ac	1	34.8	20.1 (57.8%)	32.9 (94.5%)	22,853	21,213	0.7886
ZT6	H3K9ac	2	34.2	20.3 (59.4%)	32.4 (94.7%)	21,628	20,188	
ZT12	H3K9ac	1	34.2	19.4 (56.7%)	31.7 (92.7%)	23,496	21,768	0.8879
ZT12	H3K9ac	2	49.5	27.4 (55.4%)	45.4 (91.7%)	24,097	22,240	
ZT18	H3K9ac	1	34.8	18.9 (54.3%)	32.7 (94.0%)	24,441	22,711	0.8677
ZT18	H3K9ac	2	26.5	14.8 (55.8%)	24.9 (94.0%)	21,585	20,251	

Table S2. RNA-seq alignment summary

Sample	Replicate	Total reads (millions)	Aligned reads (millions)	Transcripts with RPKM>1	R-squared transcript RPKMs
Control	1	22.9	19.9 (86.7%)	22,006	0.9523
Control	2	22.9	20.0 (87.5%)	21,852	
Starvation	1	23.5	21.0 (89.2%)	21,608	0.9244
Starvation	2	23.6	20.3 (86.1%)	21,894	
Submergence	1	22.0	19.5 (88.5%)	21,105	0.8390
Submergence	2	22.7	20.7 (91.1%)	20,984	
ZT0	1	25.6	23.4 (91.4%)	21,881	0.8471
ZT0	2	29.9	27.5 (92.0%)	22,526	
ZT6	1	30.7	28.7 (93.5%)	22,482	0.9184
ZT6	2	29.7	27.4 (92.3%)	22,007	
ZT12	1	31.2	28.8 (92.3%)	23,117	0.7955
ZT12	2	35.0	32.2 (92.0%)	23,196	
ZT18	1	36.2	33.7 (93.1%)	23,273	0.6399
ZT18	2	37.5	34.7 (92.5%)	23,598	

Table S3. GO enrichment of starvation affected genes

	GO item	all genes	expression up		expression down		K9ac up		K9ac down		Kbu up		Kbu down		Kcr up		Kcr down	
			gene number	FDR	gene number	FDR	gene	FDR	gene	FDR	gene number	FDR	gene number	FDR	gene number	FDR	gene number	FDR
GO:0006091	generation of precursor metabolites and energy	325	8	0.52943	48	3E-30	9	0.93279	21	0.93722	5	0.16281	10	0.95308	2	0.86378	3	0.83081
GO:0006629	lipid metabolic process	981	57	0.00023	28	0.90731	26	0.93855	53	0.56821	2	0.62332	24	0.52251	3	0.99896	5	0.92673
GO:0006950	response to stress	3582	188	3.4E-09	118	0.35893	107	0.29912	236	0.53425	25	0.68826	127	0.14409	15	0.82109	20	0.7787
GO:0008152	metabolic process	7362	353	4.2E-13	263	0.0014	211	0.20753	471	0.55086	34	0.68236	271	0.00327	25	0.99135	37	0.82329
GO:0009056	catabolic process	1443	69	0.01278	34	0.35489	36	0.95993	58	0.00824	6	0.80759	26	0.03793	1	0.48763	4	0.78103
GO:0009607	response to biotic stimulus	1056	67	2.8E-06	46	0.02998	32	0.6713	95	0.00242	9	0.67665	51	0.00709	7	0.52585	10	0.34195
GO:0009628	response to abiotic stimulus	2168	105	0.00076	106	5.7E-07	68	0.28627	155	0.218	19	0.30158	79	0.18771	15	0.14478	8	0.74708
GO:0009719	response to endogenous stimulus	1463	93	1.2E-08	43	0.99588	60	0.00664	123	0.00468	12	0.60656	51	0.51288	6	0.91949	8	0.84806
GO:0015979	photosynthesis	231	2	0.09424	63	5.6E-81	9	0.55037	31	0.00038	7	2.5E-05	8	0.90903	3	0.19709	3	0.80188
GO:0009579	thylakoid	553	10	0.1029	127	3E-137	17	0.80606	67	5.2E-06	13	1.2E-06	21	0.50783	8	0.00101	8	0.03651
GO:0019825	oxygen binding	345	27	0.00012	21	0.00511	11	0.78773	30	0.23324	3	0.84307	27	2.7E-05	3	0.5617	2	0.91411
GO:0003700	sequence-specific DNA binding transcription factor activity	1769	79	0.03457	54	0.85076	72	0.00339	149	0.00122	16	0.31807	68	0.14027	11	0.45727	11	0.80606

P<0.01 in red

Table S4. GO enrichment of submergence affected genes

	GO item	all genes	expression up		expression down		K9ac up		K9ac down		Kbu up		Kbu down		Kcr up		Kcr down	
			gene number	FDR	gene number	FDR	gene	FDR	gene	FDR	gene number	FDR	gene number	FDR	gene number	FDR	gene number	FDR
GO:0006139	nucleobase, nucleoside, nucleotide and nucleic acid metabolic process	3579	54	0.70256	86	0.03148	51	0.66341	88	0.3045	9	0.49546	38	0.05148	1	0.29614	18	0.0243
GO:0006950	response to stress	3582	92	0.0001	93	0.00307	64	0.0442	86	0.36058	20	0.54045	37	0.06093	12	0.45379	14	0.45245
GO:0008152	metabolic process	7362	180	1.5E-08	145	0.42855	117	0.05707	149	1.0013	41	0.46675	53	0.80193	19	0.7586	12	0.43867
GO:0009058	biosynthetic process	4643	77	0.94213	119	0.00058	65	0.66676	119	0.04987	19	0.96983	45	0.06592	6	0.98119	17	0.42949
GO:0009607	response to biotic stimulus	1056	38	2.1E-05	33	0.01033	18	0.46415	32	0.15238	6	0.89212	11	0.44802	3	1.03927	7	0.09569
GO:0009628	response to abiotic stimulus	2168	50	0.06928	56	0.03445	41	0.06398	44	1.00115	10	0.95006	21	0.37464	7	0.77271	2	0.58861
GO:0009719	response to endogenous stimulus	1463	40	0.01017	53	8.7E-06	30	0.06268	52	0.00146	7	0.97339	16	0.37576	4	1.01845	4	0.9622
GO:0030246	carbohydrate binding	234	9	0.05808	5	0.84149	6	0.28135	3	0.76645	5	0.0051	1	0.851	4	1.4E-05	0	0.78263
GO:0003700	sequence-specific DNA binding transcription factor activity	1769	44	0.03711	72	6.3E-11	41	0.00234	71	2E-07	5	0.83691	32	1.8E-07	0	0.53651	17	5.2E-08

P<0.01 in red.

Table S5. Primer sequence and annotation of genes tested in Figure S2 and S4

gene id	primer type	primer sequence F	primer sequence R	annotation
LOC_Os01g13130	RT-qPCR	TGGGTCTACTGGGTCGG	CTCCACGTCCATGAACAGG	aquaporin protein
LOC_Os01g39860	RT-qPCR	GAGGTGAAGAGCTTCGTGTAC	TGTTCAGGTGGAGGTTCTTG	1-aminocyclopropane-1-carboxylate oxidase protein
LOC_Os01g46370	RT-qPCR	CGCTGGTGAACAAGAATGTG	CCCTCGTAATCCATCAACTTCC	lipase class 3 family protein
LOC_Os01g47080	RT-qPCR	CGAGCAGAAGTTTCAGACATTG	CATAGTGGCTTCAGTTCTCAGG	pyruvate kinase
LOC_Os01g65580	ChIP-qPCR	TCGTATATCTGCTCATCTATTGGC	AATTCCCCTCCAACTCCTTG	mitotic checkpoint family protein
LOC_Os01g66080	ChIP-qPCR	AATTTAGCTGCCGAGACCTG	CGATCAGACGAAACCACAAATG	retrotransposon protein
LOC_Os01g66300	ChIP-qPCR	GGCCTCCACACCATATCTTG	GAAAGACCAAGAAACCAAGAACC	KH domain containing protein
LOC_Os02g52650	RT-qPCR	AAGAATGGGAGGTTGGCTATG	TGAACGGGTCTGAAAGATGTG	chlorophyll A-B binding protein
LOC_Os02g52650	ChIP-qPCR	GCCATGCTTCTGCTCAAAG	GCAACCAAAATCTTACAGCGG	chlorophyll A-B binding protein
LOC_Os05g40420	RT-qPCR	AGGCACTCTTCATCTTATTGGG	CCAAAACATCACGTCCATCAG	2,3-bisphosphoglycerate-independent phosphoglycerate mutase
LOC_Os07g47670	ChIP-PCR	GAAATGGCGGAGGAGAAGAG	ATGTATGAGAAAGCGATGAGAGG	hypoxia-responsive family protein
LOC_Os08g36480	RT-qPCR	ACTACCATTACAAGGACAACCG	ATCACCGAGTTCACGTTCAG	nitrate reductase
LOC_Os09g11460	RT-qPCR	GACTCGCGCATACTCATCG	GTGGTCATGTCGAAGGCG	AP2 domain containing protein
LOC_Os09g11460	ChIP-qPCR	CACTTGTCACTACCACGGATC	CTTCCGCGAGTTCCTCTC	AP2 domain containing protein
LOC_Os09g26660	RT-qPCR	TCCCACAATTTCTCCCTTCG	TGTCCAGTCTCCGTTTGTTC	ferric reductase
LOC_Os11g45400	RT-qPCR	GGGAGATGAAGGTGATTTGGG	GGTAGAGAAGCCCACAAC	glycerol-3-phosphate acyltransferase
LOC_Os12g08770	RT-qPCR	GTTCCCCTACAACTTCACCG	GCACTTGTACTTCTCCTTCCC	photosystem I reaction center subunit N, chloroplast precursor
LOC_Os12g19470	RT-qPCR	CGATCTAAGTGGGTGCCTTG	GTATCCGGGTGATCTGTGATTC	ribulose bisphosphate carboxylase small chain, chloroplast precursor
LOC_Os12g37419	ChIP-qPCR	ACAAACACTTCTAGCCTTATGGT	CGTTTGTGGCTGTGTCTTTC	cytochrome c oxidase polypeptide Vc

Table S6. Primers used in OsSRT2 mutagenesis

SRT2-PGEX4T-1-F	CCGCGTGGATCCCCGGAATTCATGGCGGCGGGGGCGCAC
SRT2-PGEX4T-1-R	CTCGAGTCGACCCGGGAATTCACTTACGTTTGGTACAGCTAGACTTCC
SRT2-S123Y-R	TTCAGTGTACATTCCTGCTCCAG
SRT2-S123Y-F	CTGGAGCAGGAATGTACACTGAA
SRT2-H215Y-R	CACACTTCCGTACAATTCAACTG
SRT2-H215Y-F	CAGTTGAATTGTACGGAAGTGTG
