# Supplementary material for: Dynamics and functional interplay of histone lysine butyrylation, crotonylation, and acetylation in rice under starvation and submergence
Source: Genome Biol. 2018 Sep 25;19:144. doi: 10.1186/s13059-018-1533-y (PMC6154804; doi:10.1186/s13059-018-1533-y)
Supplement: Supplementary file 5 — Review history. (DOCX 37 kb) [file 13059_2018_1533_MOESM5_ESM.docx]

**First round of review**

**Reviewer 1**

**Are the methods appropriate to the aims of the study, are they well described, and are necessary controls included?**
Yes

**Are the conclusions adequately supported by the data shown?**

Yes

**Are sufficient details provided to allow replication and comparison with related analyses that may have been performed?**

Yes

**Does the manuscript adhere to the journal's guidelines on** [Minimum Standards Reporting Checklist](https://resource-cms.springer.com/springer-cms/rest/v1/content/7117202/data/v2/Minimum+standards+of+reporting+checklist)**?**

Yes

**Does the work represent a significant advance over previously published studies? Please explain.**

Yes

**Is the paper of broad interest to others in the field, or of outstanding interest to a broad audience of biologists?**

Yes: epigenetics mechanism should be of interest in non-plant biologists

**Comments to authors**

In this manuscript, the authors identified histone Kbu and Kcr modifications in rice and studied genome-wide distributions of Kbu, Kcr as well as H3K9ac. Furthermore, the authors compared genome-wide changes of different histone acylations in response to stresses such as starvation and submergence, and correlate with differential gene expression through ChIP-seq and RNA-seq analysis. Finally, they tested histone deacylation activities of rice HDACs and indicated different histone acylation might be removed by different mechanisms.
This manuscript is of much interest considering the dynamic nature of plant metabolism and the fact that little is known about the roles of various metabolite-derived histone acylations in plant. The ChIP-seq and RNA-seq data could also be useful resources for future studies. However, I have some comments on the bioinformatic analysis and suggestions before publication.

Major points:
1. The authors claimed that more than 5000 genes were marked only by Kbu or Kcr. However, this analysis is based on ChIP-seq studies comparing pan anti-Kbu or anti-Kcr versus one site (H3K9ac) of Kac. It is too far to tell that these 5000 genes are marked exclusively by Kbu and Kcr but not Kac. Moreover, the affinity efficiency of different antibodies should be considered as well. To study this, in ideal situation, antibodies targeting different acylations on the same site should be used in ChIP-seq. If the authors do not have data, some explanation should is suggested. In addition, qPCR validation is necessary to compare positive genes and negative genes with each antibody simultaneously, for some example genes. 
2. Page 7, it is not correct to say that "few genes showed concurrent changes of H3K9ac and Kbu or Kcr after starvation or submergence" in Fig. 4a. Actually, there are more common genes between H3K9ac and Kbu than ones between Kbu and Kcr. 
3. Are there any pathways enriched in the non-redundant genes associated exclusive increase or decrease in Kbu, Kcr, and Kac shown in Fig. 4a?
4. The plots showing correlation in Fig. 5a does not qualify as a positive correlation with Pearson's R=0.314. Usually, a correlation is considered as weak when Pearson's R is between -0.45 and 0.45. In addition, correlation assays using transcriptome or other big data sets, p-value could be artifactual because of large number (n) of subjects. 
5. Why did the authors choose stress induced other than suppressed genes in the analysis in Fig. 5B; there are significantly more genes with decrease of Kbu than with increase of Kbu.
6. It is hard to see any difference in histone Kbu or Kcr between control and "debutyrylase/decrotonylase" mutants, but substantial change in H3K9ac. Are there any changes on specific histone sites of Kbu or Kcr in proteomics screening experiments?

Minor points:
1. Fig.1. There is strong signal for both anti-Kbu and anti-Kcr referring to histone H3 at 15Kd, as shown in Fig.1a. However, the largest number of either Kbu or Kcr sites were identified in histone H2B, as shown in Fig. 1b. These results are contradictory as what the signal from the pan antibodies represent for. A higher resolution of western blot as well as coomassie blue staining which can separate well each of the four core histones are necessary.
2. Did the authors examine the global levels of histone Kbu, Kcr, and Kac in response to starvation and submergence, by Western blot of Mass spectrometry-based quantification? 
3. Typos in page 3, "histone lycine acylations" and "crotony-CoA"
4. Page 5, the statement that "specific lysine acylation sites compared to mammalian histones" is not accurate. Not detected in one experiment does mean not existed.
5. Fig. 3B. Why is there H3K9ac enrichment at TSSs of "only Kbu and Kcr genes"?

**Reviewer 2**

**Are the methods appropriate to the aims of the study, are they well described, and are necessary controls included?**

The manuscript titled “Dynamics and functional interplay of histone lysine butyrylation, crotonylation, and acetylation in rice under starvation and submergence” by Lu et al. studies protein acylaton (butyrylation and crotonylation) in rice. Specifically they showed that rice histones are acylated using a pan-butyryl antibody and a pan-crotonyl antibody. Using the same antibodies, they profiled the whole genome distribution of the two modifications and their changes in response to two types of stresses, starvation and submergence. The changes in Kbu and Kcr were correlated with changes in mRNA levels, shown by mRNA-seq. Based on these results the authors made the following conclusions: 1. Kbu and Kcr are robust histone modifications in rice. 2. Kbu and Kcr react differently to environmental cues compared to H3K9ac. 3. SRT2 is involved in the removal of Kcr. My main concern is with the interpretation of some of the data, which need to be addressed to make the conclusions more convincing.

Technical issue:
Specificity of the pan-butyryl and pan crotonyl antibody used. Butyrylation and crotonylation are similar in composition. If cross reactivity exists between the two antibodies, that may need to the observed high percentage of overlap between the two modifications on the genome.

**Are the conclusions adequately supported by the data shown?**

1. Kbu and Kcr distribution on the chromatin are interpreted as histone Kbu and Kcr. However it was recently shown that crotonylation can be detected on 637 proteins in tobacco (Sun H. et al. 2017 Sci Rep). If a similar number of proteins are crotonylated in rice, Kcr actually profiles the distribution of crotonylated proteins (instead of histones) on the chromatin. 
2. The function of Kbu in poising gene activation by external stresses. I don’t quite understand the logic in making this conclusion. If the genes listed in figure 5B and 5C are already up-regulated, how are they poised? Following the same logic H3K9ac is involved in poising gene activation by internal cues (CR).
3. Kcr level increases in the srt2 mutant is consistent with SRT2 being a deacylase, but in vitro assays are need to show that rice SRT2 indeed has this activity. 

A minor point:
Page 5. In the 1st paragraph, it was stated that “many are at different lysine residues compared to those detected in rice.”, while earlier in the paragraph, “most of which (K9cr, K18cr, K23cr, and K27cr of H3 and K8cr, K12cr, and K16cr of H4) were also identified in human and mouse cells”. Is this because plant H2A and H2B sequences are actually very different from mammalian histones?
Some typos: 
Page 3, paragraph 2, line 5: lycine should be lysine.
Page 3, paragraph 2, line 1: histine should be histone.

**Are sufficient details provided to allow replication and comparison with related analyses that may have been performed?**

Yes

**Does the manuscript adhere to the journal's guidelines on** [Minimum Standards Reporting Checklist](https://resource-cms.springer.com/springer-cms/rest/v1/content/7117202/data/v2/Minimum+standards+of+reporting+checklist)**?**

Yes

**Is the paper of broad interest to others in the field, or of outstanding interest to a broad audience of biologists?**

Yes: Protein acylation was identified in mammals as one of the major PTMs. Their function and the function of histone acylation in particular affects our understanding of basic cellular processes such as transcriptional regulation.

**Authors’ response to reviewers**

***Reviewer #1****:*

In this manuscript, the authors identified histone Kbu and Kcr

modifications in rice and studied genome-wide distributions of Kbu, Kcr as well as

H3K9ac. Furthermore, the authors compared genome-wide changes of different

histone acylations in response to stresses such as starvation and submergence, and

correlate with differential gene expression through ChIP-seq and RNA-seq analysis.

Finally, they tested histone deacylation activities of rice HDACs and indicated different

histone acylation might be removed by different mechanisms.

This manuscript is of much interest considering the dynamic nature of plant metabolism

and the fact that little is known about the roles of various metabolite-derived histone

acylations in plant. The ChIP-seq and RNA-seq data could also be useful resources for

future studies. However, I have some comments on the bioinformatic analysis and

suggestions before publication.

Major points:

1. The authors claimed that more than 5000 genes were marked only by Kbu or Kcr.

However, this analysis is based on ChIP-seq studies comparing pan anti-Kbu or anti-

Kcr versus one site (H3K9ac) of Kac. It is too far to tell that these 5000 genes are

marked exclusively by Kbu and Kcr but not Kac. Moreover, the affinity efficiency of

different antibodies should be considered as well. To study this, in ideal situation,

antibodies targeting different acylations on the same site should be used in ChIP-seq.

If the authors do not have data, some explanation should is suggested. In addition,

qPCR validation is necessary to compare positive genes and negative genes with each

antibody simultaneously, for some example genes.

RE: Thank you for the comments to which we totally agree. Indeed, these 5000 genes

might have histone Kac at other sites and may be resulted from different antibody

affinities. We have changed wording and description in the text. Presently we do not

have H3K9bu or H3K9cr ChIP-seq data. As you suggested, we have performed anti-

Kbu/Kcr ChIP-qPCR validations of the ChIP-seq data to compare positive and negative

genes (Figure S2).

2. Page 7, it is not correct to say that "few genes showed concurrent changes of

H3K9ac and Kbu or Kcr after starvation or submergence" in Fig. 4a. Actually, there are

more common genes between H3K9ac and Kbu than ones between Kbu and Kcr.

RE: We have changed wording.

3. Are there any pathways enriched in the non-redundant genes associated exclusive

increase or decrease in Kbu, Kcr, and Kac shown in Fig. 4a?

RE: The pathways enriched in the non-redundant Kbu and Kac genes are similar to

those with changes of all modifications. Genes with exclusive Kcr changes have no

clear pathway enrichment (probably because of the small number of the genes).

4. The plots showing correlation in Fig. 5a does not qualify as a positive correlation

with Pearson's R=0.314. Usually, a correlation is considered as weak when Pearson's

R is between -0.45 and 0.45. In addition, correlation assays using transcriptome or

other big data.

RE: We agree. We have changed wording.

5. Why did the authors choose stress induced other than suppressed genes in the

analysis in Fig. 5B; there are significantly more genes with decrease of Kbu than with

increase of Kbu.

RE: Since genes marked by only Kbu or Kcr under normal conditions have relatively

low expression levels, we chose to analyze those that are stress-inducible rather than

repressed.

In Fig. 5B we intended to show whether Kbu/Kcr have a function to poise gene

induction by starvation/submergence. The groups of genes in Fig. 5B are classified by

their modification status under normal conditions.

6. It is hard to see any difference in histone Kbu or Kcr between control and

"debutyrylase/decrotonylase" mutants, but substantial change in H3K9ac. Are there

any changes on specific histone sites of Kbu or Kcr in proteomics screening

experiments?

RE: Unfortunately we do not have proteomic analysis of specific Kbu/Kcr sites in the

mutants at this stage. We have performed in vitro enzyme activity tests to support the

decrotonylase activity of OsSRT2 (Fig. 6B).

Minor points:

1. Fig.1. There is strong signal for both anti-Kbu and anti-Kcr referring to histone H3

at 15Kd, as shown in Fig.1a. However, the largest number of either Kbu or Kcr sites

were identified in histone H2B, as shown in Fig. 1b. These results are contradictory as

what the signal from the pan antibodies represent for. A higher resolution of western

blot as well as coomassie blue staining which can separate well each of the four core

histones are necessary.

RE: We have repeated the WB experiments with anti- Kbu and Kcr antibodies to

improve resolutions. Besides a band around 15 kDa (likely H3), additional bands were detected. Coomassie staining of the gel is now shown in Fig 1a.

2. Did the authors examine the global levels of histone Kbu, Kcr, and Kac in

response to starvation and submergence, by Western blot of Mass spectrometry-based

quantification?

RE: As no global change of histone Kbu, Kcr, and Kac in response to starvation and submergence was detected by ChIP-seq (the number of peaks and modified genes are similar among different groups), Western-blotting that is less sensitive than ChIP-Seq may not be appropriate or useful to detect any clear changes in this case.

3. Typos in page 3, "histone lycine acylations" and "crotony-CoA"

RE: Corrected

4. Page 5, the statement that "specific lysine acylation sites compared to mammalian

histones" is not accurate. Not detected in one experiment does mean not existed.

RE: Thank you for the point. We have deleted the statement at the end the paragraph.

5. Fig. 3B. Why is there H3K9ac enrichment at TSSs of "only Kbu and Kcr genes"?

RE: The H3K9ac peaks in Kbu/Kcr-only genes are below the threshold (p- value =1e-5 cutoff) and should not be considered as enriched.

***Reviewer #2****:*

The manuscript titled “Dynamics and functional interplay of histone lysine butyrylation, crotonylation, and acetylation in rice under starvation and submergence” by Lu et al. studies protein acylaton (butyrylation and crotonylation) in rice. Specifically they showed that rice histones are acylated using a pan-butyryl antibody and a pancrotonyl antibody. Using the same antibodies, they profiled the whole genome distribution of the two modifications and their changes in response to two types of stresses, starvation and submergence. The changes in Kbu and Kcr were correlated with changes in mRNA levels, shown by mRNA-seq. Based on these results the authors made the following conclusions: 1. Kbu and Kcr are robust histone modifications in rice. 2. Kbu and Kcr react differently to environmental cues compared to H3K9ac. 3. SRT2 is involved in the removal of Kcr. My main concern is with the interpretation of some of the data, which need to be addressed to make the conclusions more convincing.

Technical issue:

Specificity of the pan-butyryl and pan crotonyl antibody used. Butyrylation and crotonylation are similar in composition. If cross reactivity exists between the two antibodies, that may need to the observed high percentage of overlap between the two modifications on the genome.

RE: We have performed dot-blot tests of pan-anti-Kbu and pan-anti-Kcr specificity, explained in the Method section, and included as Fig S8.

Kbu and Kcr distribution on the chromatin are interpreted as histone Kbu and Kcr. However it was recently shown that crotonylation can be detected on 637 proteins in tobacco (Sun H. et al. 2017 Sci Rep). If a similar number of proteins are crotonylated in rice, Kcr actually profiles the distribution of crotonylated proteins (instead of histones) on the chromatin.

RE: Thanks for the comments. Yes, it is not excluded that some of the ChIP fragments are bound by Kbu or Kcr-modified chromatin proteins other than histones. We have stated this possibility in the text.

The function of Kbu in poising gene activation by external stresses. I don’t quite understand the logic in making this conclusion. If the genes listed in figure 5B and 5C are already up-regulated, how are they poised? Following the same logic H3K9ac is involved in poising gene activation by internal cues (CR).

RE: In Fig 5B the different groups of modified genes are defined by their modification status in normal conditions, so they are not already up-regulated, but have the potential to be induced by stresses.

Kcr level increases in the srt2 mutant is consistent with SRT2 being a deacylase, but in vitro assays are need to show that rice SRT2 indeed has this activity.

RE: We have performed in vitro tests of OsSRT2 decrotonylase activity. The data are presented in Figure 6B.

A minor point:

Page 5. In the 1st paragraph, it was stated that “many are at different lysine residues compared to those detected in rice.”, while earlier in the paragraph, “most of which (K9cr, K18cr, K23cr, and K27cr of H3 and K8cr, K12cr, and K16cr of H4) were also identified in human and mouse cells”. Is this because plant H2A and H2B sequences are actually very different from mammalian histones?

RE: thanks for the point. We have deleted the statement at the end in this paragraph.

**Second round of review**

**Reviewer 1**

The authors have adequately addressed my concerns. I would suggest to accept the paper as it is.

**Reviewer 2**

In the revised manuscript, Lu et al. performed additional experiments to address some of the questions. Notably, dot plots were used to show the specificity of pan-Kbu and pan-Kcr antibodies used; in vitro assays were performed to demonstrate the biochemical activity of OsSRT2. The manuscript is improved compared to the previous version. However I do feel that the analyses are somewhat limited, though a lot of data were generated for this study. I also have questions on the interpretation of some important results, which would affect the main conclusions of this study. 

1. “functional interplay of histone lysine butyrylation, crotonylation and acetylation”. The authors set out to study how histone acylation and acetylation “interact” to regulate gene expression, but not much analyses was performed to address this question. In the discussion the authors state that “the proportional mixture of distinct acylations” may be important for its function. This can be tested with the data present. It makes more sense to compare Kbu, Kcr and Kac quantitatively instead of qualitatively (e.g. using venn diagram; Figure 3A). How are genes containing 50% K9ac and 50% Kcr different from genes with 10% K9ac and 90% Kcr? The observation that genes marked by K9ac+Kcr+Kbu have the highest expression levels (Figure 3B) may be simply because they have the highest levels of total acylation? Following the same logic, how do the proportions of distinct acylations change quantitatively in response to subemergence and starvation? 

2. “In rice under starvation and subemergence Kbu and Kcr appeared to be less dynamic” compared to H3K9ac. This conclusion is mainly based on the observation that less up- and down-regulated regions were identified for Kbu and Kcr compared to K9ac under subemergence and starvation (figure 4A). However many factors can contribute to the results: a mixture of signals (cr or bu on different lysines) may change less compared to H3K9ac (a single modification); Kbu/Kcr/H3K9ac may be regulated at different time points after the stress. Without more information on how each modification changes over time, the dynamics of each modification cannot be deduced. 

3. Figure 6B and 6C. The in vitro activities of OsSRT2 were only 2-fold of the background. To make sure that the observed decrotonylase activity is indeed due to SRT2 (instead of other proteins copurified with SRT2), point mutations in the active site of SRT2 need to be used as controls. 

A minor point: 
Page 6. Paragraph 3. “The substrates for histone acetylation/acylatin … are important cellular primary metabolites. Stress conditions such as … affect greatly plant primary metabolism.” Are there references showing that acyl-CoA levels are affected under these stressed conditions? The possibility that these stresses affect chromatin modifications and gene expression through other means should be mentioned. In addition a related study (Chen et al. 2017 Nat Plants) showed that increased cytosolic acetyl-CoA levels may only promote specific histone modifications (H3K27ac) instead of acetylation in general. 

Typo? 
Abstract/Results: line1 “we identified histones 4 lysine butyrylation”

**Authors’ response to reviewers**

***Reviewer #2****:*

In the revised manuscript, Lu et al. performed additional experiments to address some of the questions. Notably, dot plots were used to show the specificity of pan-Kbu and pan-Kcr antibodies used; in vitro assays were performed to demonstrate the biochemical activity of OsSRT2. The manuscript is improved compared to the previous version. However I do feel that the analyses are somewhat limited, though a lot of data were generated for this study. I also have questions on the interpretation of some important results, which would affect the main conclusions of this study.

1. “functional interplay of histone lysine butyrylation, crotonylation and acetylation”. The authors set out to study how histone acylation and acetylation “interact” to regulate gene expression, but not much analyses was performed to address this question. In the discussion the authors state that “the proportional mixture of distinct acylations” may be important for its function. This can be tested with the data present. It makes more sense to compare Kbu, Kcr and Kac quantitatively instead of qualitatively (e.g. using venn diagram; Figure 3A). How are genes containing 50% K9ac and 50% Kcr different from genes with 10% K9ac and 90% Kcr? The observation that genes marked by K9ac+Kcr+Kbu have the highest expression levels (Figure 3B) may be simply because they have the highest levels of total acylation? Following the same logic, how do the proportions of distinct acylations change quantitatively in response to subemergence and starvation?

RE: Thanks for the advice. We have compared quantitatively Kbu/Kcr/H3K9ac in the marked genes and their relationship with gene expression. H3K9ac proportion of the total acylation (i.e. Kbu/Kcr/K9ac) varies in a wider range among the marked genes than that of Kbu or Kcr (Fig 3C). Genes with higher proportion of H3K9ac are highly expressed while genes with high proportion of Kbu/Kcr have lower expression levels (Fig 3E). These results are consistent with our analysis showing that H3K9ac is more dynamic and predominant in gene activity.

We have analyzed quantitative changes of Kbu, Kcr and H3K9ac proportions in the DEGs under submergence and starvation (Fig 5B). In upregulated DEGs, H3K9ac proportion increases whereas Kbu or Kcr proportion decreases. In downregulated DEGs, we observed the reversed trend. This confirms that H3K9ac plays a predominant role in gene activation in stress response.

Our analysis of the total acylation indicates that the K9ac+Kcr+Kbu-marked genes show no higher total acylation level than those marked by K9ac+Kbu or K9ac+Kcr only (Fig 3F). Therefore, it is unlikely that the total acylation level plays a role in gene activation.

2. “In rice under starvation and subemergence Kbu and Kcr appeared to be less dynamic” compared to H3K9ac. This conclusion is mainly based on the observation that less up- and down-regulated regions were identified for Kbu and Kcr compared to K9ac under subemergence and starvation (figure 4A). However many factors can contribute to the results: a mixture of signals (cr or bu on different lysines) may change less compared to H3K9ac (a single modification); Kbu/Kcr/H3K9ac may be regulated at different time points after the stress. Without more information on how each modification changes over time, the dynamics of each modification cannot be deduced.

RE: Thanks for the points. We have included discussion of those possibilities at the

end of Discussion.

3. Figure 6B and 6C. The in vitro activities of OsSRT2 were only 2-fold of the background. To make sure that the observed decrotonylase activity is indeed due to SRT2 (instead of other proteins copurified with SRT2), point mutations in the active site of SRT2 need to be used as controls.

RE: We have performed point mutations S123Y and H215Y in the OsSRT2 catalytic domain. The Ser and His residues are conserved in plant and human sirtuins. The mutated proteins were used in the in vitro activity test in Figure 6B.

In addition, the two-fold activity of OsSRT2 that we observed in in vitro assays is comparable to published decrotonylase activity of sirtuin proteins in animal cells.

minor point:

Page 6. Paragraph 3. “The substrates for histone acetylation/acylatin … are important cellular primary metabolites. Stress conditions such as … affect greatly plant primary metabolism.” Are there references showing that acyl-CoA levels are affected under these stressed conditions? The possibility that these stresses affect chromatin modifications and gene expression through other means should be mentioned. In addition a related study (Chen et al. 2017 Nat Plants) showed that increased cytosolic acetyl-CoA levels may only promote specific histone modifications (H3K27ac) instead of acetylation in general.

RE: We have now discussed the point in the text.

Actually we have difficulty to understand why increases of cytosolic acetyl CoA (by transgenics) only promote H3K27ac through GCN5 in Chen et al. 2017, while this HAT is involved mostly H3K14 and H3K9 acetylation by several labs.

Typo?

Abstract/Results: line1 “we identified histones 4 lysine butyrylation”

RE: changed
